# A journey into the regulatory secrets of the *de novo* purine nucleotide biosynthesis

**DOI:** 10.3389/fphar.2024.1329011

**Published:** 2024-02-20

**Authors:** Nour Ayoub, Antoine Gedeon, Hélène Munier-Lehmann

**Affiliations:** ^1^ Institut Pasteur, Université Paris Cité, INSERM UMRS-1124, Paris, France; ^2^ Sorbonne Université, École Normale Supérieure, Université PSL, CNRS UMR7203, Laboratoire des Biomolécules, LBM, Paris, France

**Keywords:** allostery, antibacterial agents, chemical compounds, enzyme regulation, IMP dehydrogenase, nucleotide biosynthesis, protein-protein interactions, protein structure-function relationship

## Abstract

*De novo* purine nucleotide biosynthesis (DNPNB) consists of sequential reactions that are majorly conserved in living organisms. Several regulation events take place to maintain physiological concentrations of adenylate and guanylate nucleotides in cells and to fine-tune the production of purine nucleotides in response to changing cellular demands. Recent years have seen a renewed interest in the DNPNB enzymes, with some being highlighted as promising targets for therapeutic molecules. Herein, a review of two newly revealed modes of regulation of the DNPNB pathway has been carried out: i) the unprecedent allosteric regulation of one of the limiting enzymes of the pathway named inosine 5′-monophosphate dehydrogenase (IMPDH), and ii) the supramolecular assembly of DNPNB enzymes. Moreover, recent advances that revealed the therapeutic potential of DNPNB enzymes in bacteria could open the road for the pharmacological development of novel antibiotics.

## 1 Introduction

Primary metabolites are essential for cellular growth and survival. During evolution, organisms initially relied on the environment to procure their nutrients and have since then acquired complex metabolic pathways to ensure maximum self-sustainability by synthesizing/degrading their own biomolecules. Metabolic pathways [see public databases such as BRENDA (Chang et al., 2020), KEGG (Kanehisa et al., 2022) and MetaCyc (Caspi et al., 2020)] of primary metabolites, defined as series of sequential chemical reactions catalyzed by enzymes that produce (anabolism) or break down (catabolism) molecules, are highly conserved and finely regulated by diverse mechanisms. Despite their importance, regulation of most metabolic pathways is not fully explored, especially in bacteria.

**FIGURE 1:**
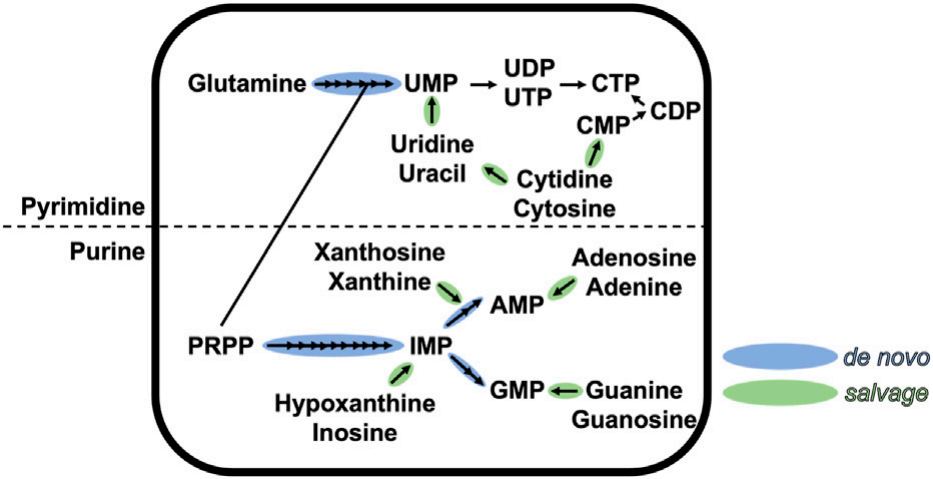
Schematic representation of the *de novo* and salvage pathways of purine and pyrimidine ribonucleotides. Each arrow represents one chemical step. This figure is not exhaustive.

Nucleotide metabolism, one of the primary metabolic pathways, plays a key role in homeostasis and cell physiology and exists in all three domains of life (Neuhard and Nygaard, 1987; Zalkin and Nygaard, 1996; Moffatt and Ashihara, 2002; Jensen et al., 2008; Lane and Fan, 2015). Nucleotides are central metabolites that are formed of three entities: i) a heterocyclic nitrogenous base, classified as purines or pyrimidines; ii) a sugar; and iii) a 5′-mono/di/tri-phosphate. All classical nucleotides share the same chemical structure, with the base linked via a β-N-glycosidic bond to the hydroxyl function of C′1 of the sugar, and the phosphate linked to the hydroxyl function of C′5 of the sugar. Nucleotides are not only building blocks used by polymerases to synthesize nucleic acids, but are also involved in multiple cellular processes, such as enzyme cofactors, metabolic precursors or signal transducers (Rudolph, 1994; Carver and Allan Walker, 1995). Because of its central position in metabolism, nucleotide pools need to be continuously replenished during highly demanding conditions, such as cell division. The pool of purine and pyrimidine nucleotides is ensured by two conserved pathways (schematized in Figure 1 for ribonucleotides): the salvage pathway that recycles free nitrogenous bases, and the *de novo* (which means “*of new*” in latin) biosynthesis pathway that produces nucleotides from carbon and nitrogen precursors (Traut, 2014). The first pathway is less demanding in cellular energy and occurs exclusively in the cytoplasm. The latter pathway is energy-consuming and can occur in the cytoplasm and possibly partially in the mitochondria/chloroplast [in the case of purine synthesis in plants or pyrimidine synthesis in animals and plants (Atkins et al., 1997)].

Since their discovery, regulation of enzymes catalyzing *de novo* purine nucleotide biosynthesis (DNPNB) by retroinhibition of end-products were minutely investigated. In the past two decades, because of their renowned potential as drug targets in anticancer and antimicrobial therapies, metabolic regulation modes of DNPNB enzymes have been revisited and investigated more thoroughly. In this review, we systematically highlight the latest advancements of DNPNB regulation in eukaryotes, and more interestingly in prokaryotes, on different levels: we describe the DNPNB organization in vertebrates and in bacteria and the different levels of regulation; we delineate the most historically studied enzyme of the pathway and an already well-known immunosuppressive target, inosine 5′-monophosphate dehydrogenase, and the multiple modes of its activity regulation via allosteric modulation, variation of oligomeric switch and formation of intracellular mesoscale filaments; we also detail the studious work on all DNPNB enzymes revealing their capacity to cluster into a supramolecular assembly (named “purinosome” in mammalian cells) specialized in purine nucleotide synthesis. Perspectives regarding the druggability of these enzymes are also highlighted, shedding the light on new possible therapeutic treatments based on DNPNB modulators. As recent reviews detailed therapeutic approaches targeting nucleotide metabolism to treat cancers and immune diseases (Wu et al., 2022; Ali and Ben-Sahra, 2023; Mullen and Singh, 2023), here we focus on the therapeutic potential of DNPNB enzymes against bacterial infections.

## 2 The *de novo* purine nucleotide biosynthesis (DNPNB)

### 2.1 General overview of the DNPNB pathway organization

**FIGURE 2:**
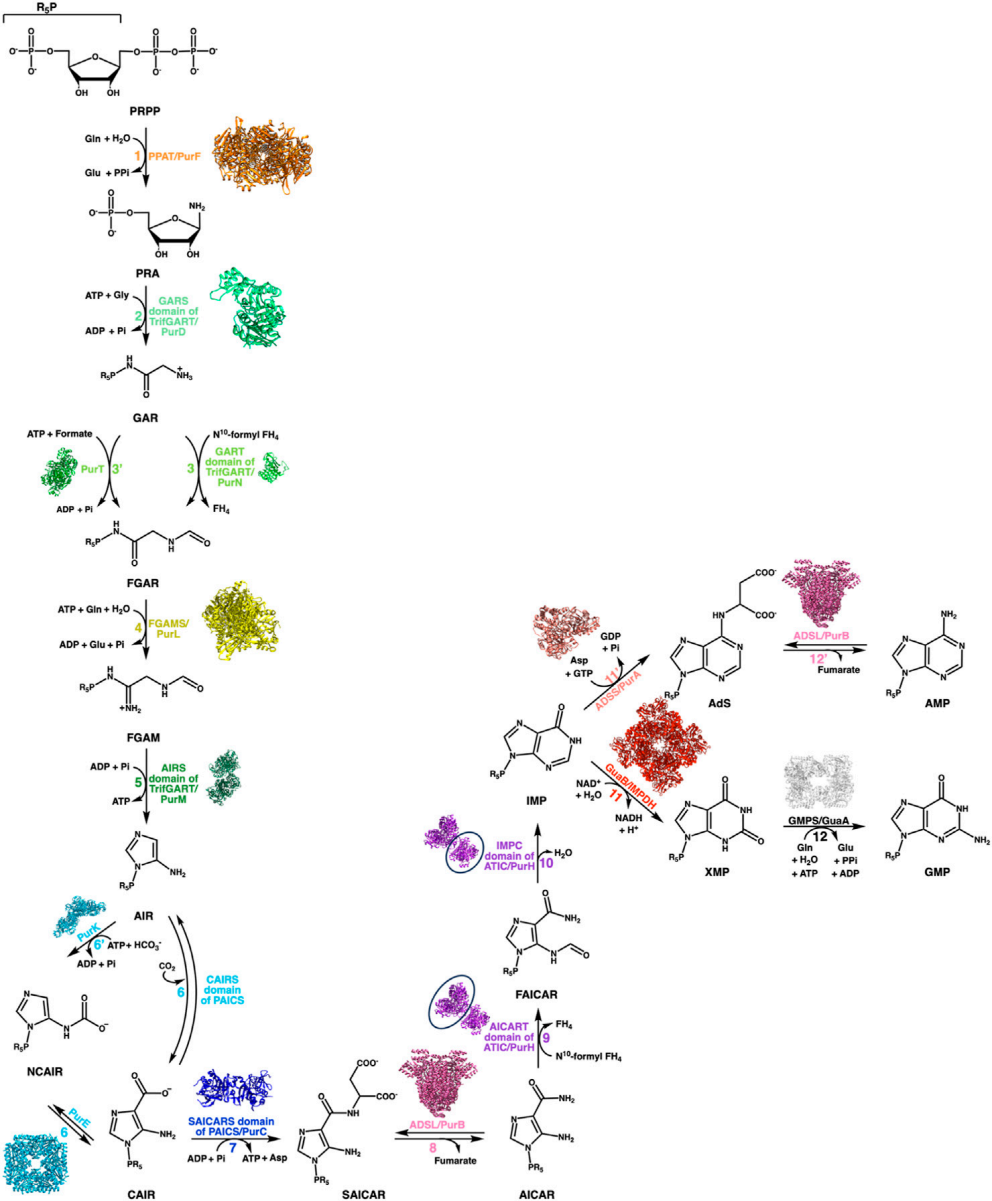
DNPNB pathway. Representation of the sequential conversion of PRPP into AMP and GMP *via* the *de novo* biosynthetic pathway of purine nucleotides. The steps are labeled with specific colors and numbered, with a corresponding list of enzymes involved in each step provided for both bacteria and vertebrates (see Table 1). In vertebrates, six enzymes are responsible for converting PRPP to IMP, which is then further processed into GMP or AMP by several additional reactions. Prokaryotes, on the other hand, require fourteen enzymes to catalyze the chemical steps. The structure of each *E. coli* enzyme is illustrated. Resolved structures of *E. coli* enzymes have the following PDB accession numbers: 1ECB (PurF), 1GSO (PurD), 1CDE (PurN), 1KJ8 (PurT); 1CLI (PurM), 1D7A (PurE), 1B6S (PurK), 2GQS (PurC), 1PTR (PurB), 1GPM (GuaA), 1ADE (PurA). For other *E. coli* enzymes to which no structure was resolved, the ortholog structures are shown with the following PDB accession numbers and percentage of sequence identities: 6JTA (*Salmonella typhimurium* PurL, 94% identity), 4A1O (*Mycobacterium tuberculosis* PurH, 43% identity), and 4DQW (*Pseudomonas aeruginosa* IMPDH, 66% identity).

**FIGURE 3:**
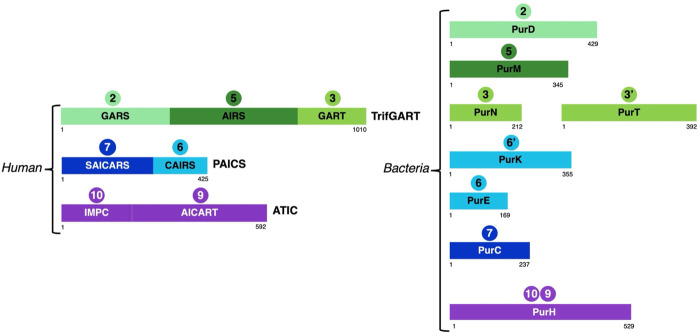
Schematic representation of the structural domains of enzymes involved in steps 2 (GARS, PurD), 3 (GART, PurN), 3'(PurT), 5 (AIRS, PurM), 6 (CAIRS, PurE), 6’ (PurK), 7 (SAICARS, PurC), 9 (AICART, PurH) and 10 (IMPC, PurH) in vertebrates and bacteria taking as representatives human and *E. coli* enzymes.

The DNPNB pathway (Figure 2) is formed of chemical steps that convert the 5-phosphoribosyl-1-pyrophosphate (PRPP) into inosine 5′-monophosphate (IMP), which is then transformed either into adenosine 5′-monophosphate (AMP) or guanosine 5′-monophosphate (GMP) (Buchanan and Hartman, 1959). The general organization of the DNPNB pathway is similar between eukaryotes and bacteria. However, some notable key differences (some highlighted in Figure 3) exist across all domains of life [see also the review by Chua and Fraser (Chua and Fraser, 2020)].

First, the number of DNPNB enzymes (see Table 1) differs between vertebrates and bacteria (nine *versus* fourteen enzymes, respectively), with the bacterial pathway being mostly dominated by monofunctional enzymes. The disparity in enzyme numbers is attributed to energetic adaptation and heterologous evolution of their catalytic domains (Zhang et al., 2008).

**TABLE 1:** Each step of the DNPNB pathway is listed with the name of the enzyme involved in the reaction, as well as its acronym for mammals and *E. coli* enzymes. The chemical steps have been sequentially numbered and given a specific color as in Figure 2. Alternative abbreviations can be found in the literature for TrifGART (tGART or GART) and FGAMS (PFAS or FGARAT).

		Abbreviation/acronym	
Step	Name of enzyme/domain and EC codes	Mammals	*E. coli*
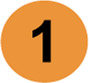	PRPP amidotransferase (2.4.2.14)	PPAT	PurF
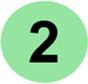	Phosphoribosylglycinamide synthetase (6.4.3.13)	GARS domain of TrifGART	PurD
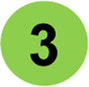	Phosphoribosylglycinamide formyltransferase (2.1.2.2)	GART domain of TrifGART	PurN
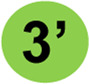	Formate-dependent phosphoribosylglycinamide formyltransferase (6.3.1.21)	—	PurT
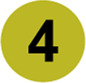	Phosphoribosyl formylglycinamidine synthase (6.3.5.3)	FGAMS	PurL
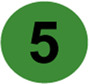	Phosphoribosylaminoimidazole synthetase (6.3.3.1)	AIRS domain of TrifGART	PurM
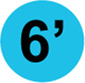	N^5^-carboxyaminoimidazole ribonucleotide synthase (6.3.4.18)	—	PurK
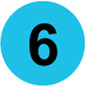	N^5^-carboxyaminoimidazole ribonucleotide mutase (5.4.99.18)	—	PurE
	Phosphoribosyl aminoimidazole carboxylase (4.1.1.21)	CAIRS domain of PAICS	-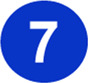	Phosphoribosyl aminoimidazole succinocarboxamide synthetase (6.3.2.6)	SAICARS domain of PAICS	PurC
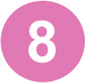	Adenylosuccinate lyase (4.3.2.2)	ADSL	PurB
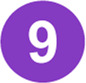	5-aminoimidazole-4-carboxamide ribonucleotide formyltransferase (2.1.2.3)	AICART domain of ATIC	PurH
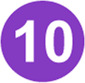	IMP cyclohydrolase (3.5.4.10)	IMPC domain of ATIC	PurH
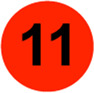	Inosine 5′-monophosphate dehydrogenase (1.1.1.205)	IMPDH1, IMPDH2	GuaB
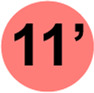	Adenylosuccinate synthase (6.3.4.4)	ADSS1, ADSS2	PurA
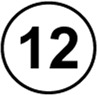	Guanosine 5′-monophosphate synthase (6.3.5.2)	GMPS	GuaA
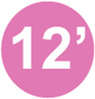	Adenylosuccinate lyase (4.3.2.2)	ADSL	PurB

Indeed, in vertebrates, six enzymes are involved for the catalysis of ten reactions leading to IMP production. Among these are two multifunctional enzymes: phosphoribosylglycinamide formyltransferase (TrifGART) and phosphoribosyl aminoimidazole carboxylase (PAICS). TrifGART is a trifunctional enzyme that consists of three catalytic domains: phosphoribosyl-amine glycine ligase (GARS), phosphoribosylglycinamide-formyl transferase (GART), and phosphoribosyl-formylglycinamide cyclo-ligase (AIRS), and is responsible for catalyzing steps 2, 3, and 5 (Daubner et al., 1985; Schrimsher et al., 1986a; Daubner et al., 1986). PAICS is a bifunctional enzyme consisting of phosphoribosyl aminoimidazole carboxylase (CAIRS) and phosphoribosyl-aminoimidazole-succinocarboxamide synthase (SAICARS), and catalyzing steps 6 and 7 (Chen et al., 1990), respectively. In bacteria, TrifGART is replaced by three prokaryotic enzymes homologous to each TrifGART domain, with PurD (Cheng et al., 1990) catalyzing step 2, PurN (Dev and Harvey, 1978; Inglese et al., 1990) catalyzing step 3, and PurM (Schrimsher et al., 1986b) catalyzing step 5 (see Figure 3).

Additionally, bacteria have an alternate reaction, denoted as step 3′, which uses ATP and formate instead of N^10^-formyl tetrahydrofolate (N^10^-formyl FH_4_). This step is catalyzed by a ligase, PurT (Marolewski et al., 1994; Marolewski et al., 1997), which has no counterpart in human.

In the case of PAICS, it is replaced by PurE [step 6; (Tiedeman et al., 1989; Mueller et al., 1994)] and PurC [step 7; (Meyer et al., 1992; Nelson et al., 2005)]. Another notable difference here is the presence of an additional reaction, referred to as step 6′, which modifies the enzyme specificity of step 6, of carboxylation of the intermediate aminoimidazole ribonucleotide (AIR) to 4-carboxy-5-aminoimidazole ribonucleotide (CAIR). The AIR molecule is then converted to N^5^-carboxyamino-imidazole ribonucleotide (NCAIR) by bicarbonate ligation by the monofunctional enzyme PurK (Tiedeman et al., 1989; Meyer et al., 1992; Mueller et al., 1994), followed by conversion to CAIR by the monofunctional mutase PurE. On the other hand, in most eukaryotes, the CO_2_-dependent carboxylation reaction occurs directly from AIR to CAIR, skipping the passage by the NCAIR intermediate. This reaction is catalyzed by the CAIRS domain of the bifunctional PAICS enzyme. This variation in the pathway seems to have an energetic advantage as the PurK-PurE pathway requires ATP, while the PAICS enzyme does not consume ATP (Zhang et al., 2008). These modifications in bacteria alter the ATP consumption, requiring five or six molecules of ATP (depending on the choice between steps 3 and 3′) and one molecule of bicarbonate instead of CO_2_.

Additionally, variations have been observed for the enzyme phosphoribosyl-formylglycinamide synthase (FGAMS in eukaryotes, PurL in bacteria), which catalyzes the fourth reaction involving the amidation of N-formylglycinamide ribonucleotide (FGAR) to N-formylglycinamidine ribonucleotide (FGAM). In eukaryotes (Mizobuchi and Buchanan, 1968a; b; Buchanan, 1982) and Gram-negative bacteria (Sampei and Mizobuchi, 1989; Schendel et al., 1989; Anand et al., 2004b), this reaction is catalyzed by a large enzyme which can be divided into a glutaminase domain that releases ammonia, a FGAM synthase domain that grafts the ammonia onto FGAR, and a N-terminal domain contacting the central FGAM synthase and glutaminase domains and probably involved in the coupling between the two active sites (Anand et al., 2004b). In contrast, in Gram-positive bacteria (Ebbole and Zalkin, 1987; Saxild and Nygaard, 2000; Anand et al., 2004a; Hoskins et al., 2004), this enzyme is replaced by three proteins: a small version of PurL, PurQ, and PurS, which are homologous to the three domains mentioned above. These proteins fit together with a 1:1:2 stoichiometry (Hoskins et al., 2004).

All other enzymes are relatively conserved (complete names and corresponding acronyms for mammals and bacteria are given in Table 1). Steps 1, 11, 11′, and 12 are catalyzed in mammals and bacteria by monofunctional enzymes: PPAT (Holmes et al., 1973) or PurF (Messenger and Zalkin, 1979; Smith et al., 1994) for step 1, IMPDH1/IMPDH2 or GuaB (see Section 3) for step 11, ADSS1/ADSS2 (Matsuda et al., 1977; Iancu et al., 2001) or PurA (Rudolph and Fromm, 1969; Bass et al., 1987; Honzatko and Fromm, 1999) for step 11’, GMPS (Nakamura and Lou, 1995; Bhat et al., 2008) or GuaA (Lee and Hartman, 1974; Patel et al., 1975; Zalkin and Truitt, 1977) for step 12. On the other hand, steps 8 and 12 as well as steps 9 and 10 are catalyzed by bifunctional enzymes: ADSL (Stone et al., 1993) or PurB (Gendron et al., 1992; Green et al., 1996; Banerjee et al., 2014) and ATIC (Rayl et al., 1996) or PurH (Aiba and Mizobuchi, 1989), respectively.

### 2.2 Regulation of the DNPNB pathway

Under the conditions of higher requirement for purine nucleotides, such as dividing cells and tumor cells, the DNPNB pathway is fundamental to replenish the purine pool. This pathway also plays a central role in bacteria. Thus, different levels of regulation have evolved to tightly control this pathway to adapt to environmental changes.

#### 2.2.1 Transcriptional regulation

At the transcriptional level, c-Myc transcription factor controls the expression of nearly 15% of the human genome, including genes involved in nucleotide biosynthesis. For example, c-Myc and its downstream target oncogene and translation initiation factor eIF4E activate the expression of the *PRPS2* gene (coding for phosphoribosyl pyrophosphate synthetase 2 involved in PRPP synthesis, first substrate of the DNPNB pathway) by controlling the cis-regulatory element in the 5′UTR of PRPP synthetase mRNA (Cunningham et al., 2014; Lane and Fan, 2015). In addition, it has been demonstrated that c-Myc plays a critical role in the maintenance of expression of PRPS2, PPAT, TrifGART, PAICS and IMPDH2 in melanoma cells (Mannava et al., 2008) and of PPAT, IMPDH1, IMPDH2 but not PAICS in a human lymphoma model cell line (Liu et al., 2008). Besides c-Myc, other oncogenes or tumor suppressors have also been shown in cancer cells to regulate the DNPNB pathway at the transcriptional level (Villa et al., 2019). More recently, in human lung cancer cells exhibiting a high level of the vestigial-like family member 3 (VGLL3), a cofactor for TEA domain transcription factors (TEADs), it has been demonstrated that TrifGART as well as PPAT (but not PAICS) expression is increased, but only *GART* gene knockdown reduces significantly cell proliferation (while *PPAT* gene knockdown has no impact), suggesting that VGLL3 stimulates the DNPNB pathway through TrifGART expression (Kawamura et al., 2022).

**FIGURE 4:**
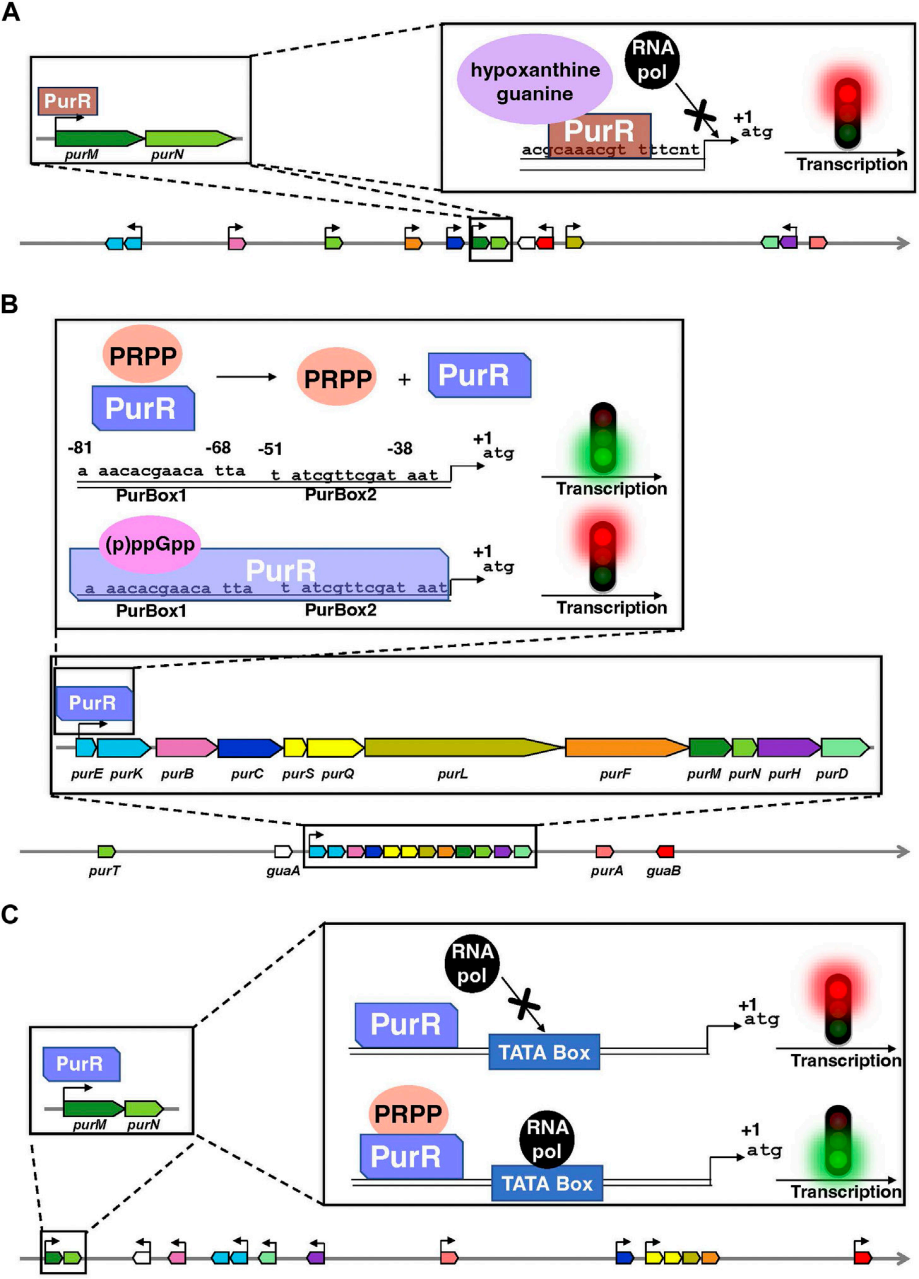
Organization of the genes encoding the enzymes of the *de novo* pathway, and their mode of transcriptional regulation by PurR in *E. coli* **(A)**, *B. subtilis* **(B)** and *L. lactis* **(C)**. Data are extracted from the BioCyc database (Caspi et al., 2020). In each panel, coding regions are shown by boxes with the same color code as in Figure 2. Horizontal arrows show the transcription direction from the promoters regulated by PurR and the +1 represents the first nucleotide transcribed. Of note, the PurR found in *E. coli* is non-homologous to the ones present in *B. subtilis* and *L. lactis*. Above the schematic diagram are detailed the regulatory elements on *purMN* operon as an example for *E. coli and L. lactis* and on the single *pur* operon for *B. subtilis.* **(A)** PurR (brown) interacts through a DNA binding sequence in the different *pur* operons or single genes and blocks the interaction of the RNA polymerase (RNA pol in black) with the TATA box, thus inhibiting the transcription. Hypoxanthine and guanine (mauve) are corepressors. **(B)** The interaction of (p)ppGpp (pink) with PurR (light cobalt blue) favors PurR binding to a DNA binding site composed of two boxes PurBox1 and PurBox2 upstream of the *pur* operon and inhibit the transcription of DNA. Conformational changes in PurR that occur upon PRPP (orange) binding inhibits PurR-DNA interaction, that results in the activation of DNA transcription. **(C)** The PurR (light cobalt blue) is in continuous interaction with DNA but only becomes activated after binding of PRPP (orange) on its allosteric site, which induces the recruitment of RNA polymerase (RNA pol in black) that binds to the TATA box of the promoter.

In bacteria, the transcription factor PurR is the primary regulatory protein of the transcription of DNPNB genes (Figure 4). In *E. coli* and *Salmonella typhimurium,* PurR plays a key role in free purine nucleotides sensing and regulates the expression of most of the *pur* genes (He et al., 1990; Meng et al., 1990; Zalkin and Nygaard, 1996). It is a dimer of 38 kDa and functions as a transcriptional repressor. It binds to PUR boxes (Tiedeman et al., 1989; Meng et al., 1990) located upstream of the *pur* [except for *purA*, which is downregulated via another transcription factor, MarA (Schneiders et al., 2004)] and *guaBA* operon (Figure 4A). Hypoxanthine and guanine are corepressors (Meng and Nygaard, 1990; Rolfes and Zalkin, 1990): their binding to the C-terminal domain leads to conformational changes in the N-terminal DNA-binding domain of PurR (Schumacher et al., 1994), favoring its interaction through a helix-turn-helix motif with the palindromic conserved sequence of the *pur* operons. This interaction induces the opening of the DNA minor groove, which tilts the operon, resulting in a loss of RNA polymerase accessibility to the promoter (Figure 4A). More recently, another transcription factor known as the ribose regulator RbsR has been identified in *E. coli*. It represses the transcription of *purHD* operon resulting in the inhibition of the synthesis of purine nucleotides. On the other hand, RbsR activates the expression of genes coding for enzymes participating in the salvage pathway: it was thus proposed to be implicated in the switch between *de novo* and salvage pathways depending on the growth conditions (Shimada et al., 2013).

A similar mode of regulation as the one described in enterobacteria has been found in *B. subtilis* and lactic acid bacteria such as *Lactococcus lactis* (Kilstrup et al., 2005). It also involves a PurR protein, which binds to the operator region of DNA and inhibits [*Bacillus subtilis*; (Weng et al., 1995)] or activates [*L. lactis*; (Kilstrup and Martinussen, 1998)] the transcription of the downstream genes (Figures 4B, C). In both cases, PurR contains a PRPP-binding site and high PRPP concentrations induce an activation of transcription (Weng et al., 1995; Kilstrup and Martinussen, 1998). On the other hand, the alarmones guanosine-3′,5′-tetraphosphate (ppGpp) and guanosine-3′-diphosphate 5′-triphosphate (pppGpp), jointly known as (p)ppGpp, have been recently shown to be allosteric effectors of PurR. (p)ppGpp are synthesized in response to nutrient limitation or other environmental stresses and regulates gene expression, metabolism, and other cellular processes (Steinchen et al., 2020; Anderson et al., 2021). In the case of PurR, (p)ppGpp compete for PRPP and thus promote PurR binding to DNA and repression of the transcription (Anderson et al., 2022).

In *B. subtilis*, the single *pur* operon consisting of twelve DNPNB genes (see Figure 4B) is also controlled by a purine riboswitch, located between the promoter and the translation start site of the first gene, *i.e., purE* (Ebbole and Zalkin, 1987; Mandal et al., 2003; Lins et al., 2022). The *purE* riboswitch turns off gene expression when bound to guanine. However, *L. lactis* and other Gram-positive bacteria (such as *Listeria* and *Staphylococcus*) have the same *pur* operon organization but does not possess an upstream purine riboswitch (Singh and Sengupta, 2012). A more widely distributed riboswitch class has been recently demonstrated to bind AICAR (also known as ZMP) and ZTP, the 5′-triphosphorylated derivative of AICAR (Kim P. B. et al., 2015). It is associated with genes for DNPNB (predominantly *purH*) and folate (including N^10^-formyl FH_4_ synthesis) metabolism. This mode of regulation makes it possible to link both pathways to respond adequately to N^10^-formyl FH_4_ deficiency in bacteria.

#### 2.2.2 Post-transcriptional regulation

In humans, another mode of regulation at the translational level *via* microRNAs (He and Hannon, 2004; Ratti et al., 2020) has been described for PAICS. miR-128 is responsible of a negative regulation of PAICS expression through its binding to the 3′UTR. In various human malignancies, miR-128 is downregulated leading to PAICS upregulation, which has been associated with different types of cancer (Goswami et al., 2015; Chakravarthi et al., 2017; Chakravarthi et al., 2018; Agarwal et al., 2020a; Agarwal et al., 2020b).

#### 2.2.3 Regulation at the protein level

**FIGURE 5:**
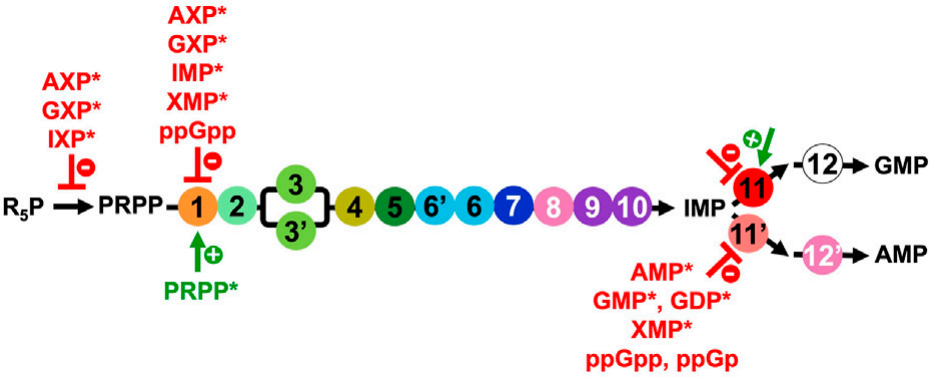
Overview of the regulators of the PRPP synthetase and DNPNB enzymes from *E. coli*, apart from IMPDH (step 11) for which the regulation will be elaborated below (Section 3.2 and list of inhibitors and activators in Table 2). NXP corresponds to 5′-mono, di or triphosphate nucleotides. Inhibitors are in red, and the only activator (PRPP) is in green. Regulators marked with an asterisk (*) were also shown to be modulators for the human counterparts.

The DNPNB pathway is also regulated at the protein level either by post-translational modifications (PTMs) or through the binding of different effectors (Figure 5).

In humans, a proteomic strategy to systematically map PTMs (acetylation, methylation, phosphorylation and ubiquitination) of the six DNPNB enzymes catalyzing IMP production has been recently performed, enabling the identification of 118 novel PTMs (Liu et al., 2019). Moreover, differences in the PTM pattern have been observed between cells grown in purine-supplemented and purine-depleted media. The authors proposed that these PTMs could play a significant role in modulating catalytic activity, interactions with other proteins, or oligomerization states, among other functions. Specifically, phosphorylation events might be of particular importance in connecting signaling pathways and cell cycle regulatory mechanisms to the DNPNB pathway. This is, for example, the case for the RAS-ERK signaling pathway. It was shown that there is no impact at the transcriptional level nor on the DNPNB enzyme levels. The stimulation of the DNPNB pathway is mediated trough the direct phosphorylation by ERK2 (but not by ERK1) of the enzyme catalyzing step 4, namely, FGAMS (Ali et al., 2020). Similarly, in *E. coli*, lysine acetylations in PurB, PurH, PurT and IMPDH enzymes have also been reported (Zhang et al., 2009).

However, the precise molecular effects of all these modifications are yet to be characterized.

Besides PTMs, the catalytic activity of DNPNB enzymes is regulated through the binding of ligands (Figure 5). The main mode of regulation of the overall rate of DNPNB is exerted through end product inhibition by purine nucleotides to maintain their appropriate levels in the cell (Zalkin and Nygaard, 1996; Nelson and Cox, 2005; Zhang et al., 2008). Three DNPNB enzymes catalyzing steps 1, 11 and 11′have been reported to be regulated by feedback inhibition (Figure 5). It starts with the first enzyme of the pathway. PurF has been demonstrated to be negatively retro-controlled by adenine and guanine nucleotides: the 5′-monophosphates are the most potent inhibitors, with AMP and GMP exhibiting a synergistic effect (Holmes et al., 1973; Messenger and Zalkin, 1979; Smith, 1998). IMP and xanthosine-5′-monophosphate (XMP) were also shown to be inhibitors. Furthermore, activation by the substrate PRPP has been observed through binding to the N-terminal glutaminase domain of the enzyme, resulting in a better affinity for glutamine and an increase of the k_cat_ (Kim et al., 1996; Bera et al., 2000). In addition, in *E. coli*, it has been demonstrated that the alarmone ppGpp is a competitive inhibitor for PurF and induces the repression of the *de novo* synthesis of nucleotides *in vitro* and *in vivo* (Wang et al., 2014). The 3D-structure of the complex of PurF with ppGpp (PDB6CZF) has revealed that ppGpp binding traps the enzyme in its inactive conformation. In this same study (Wang et al., 2014), two other ppGpp protein binders belonging to the DNPNB pathway, namely, PurC and PurB, have been identified, but they were not validated by biochemical assays. Regarding the two enzymes catalyzing the reactions that consume IMP, several modulators have been described. For PurA (step 11′), different purine nucleotides have been described as inhibitors: AMP and XMP as competitive with respect to IMP, and GMP and GDP as competitive with respect to GTP (Rudolph and Fromm, 1969; Gallant et al., 1971; Van der Weyden and Kelly, 1974; Stayton et al., 1983). Furthermore, ppGpp and guanosine-3′-monophosphate- 5′-diphosphate (ppGp) are the most potent inhibitors of *E. coli* PurA (Gallant et al., 1971; Stayton and Fromm, 1979; Pao and Dyess, 1981). However, conflicting information regarding the inhibition mechanism can be found in the literature: ppGpp is either a competitive or a non-competitive inhibitor with respect to the substrates GTP or IMP (Gallant et al., 1971; Stayton and Fromm, 1979; Pao and Dyess, 1981). Concerning IMPDH (step 11), data are presented in a dedicated section (see Section 3.2 and Table 2).

**TABLE 2:** Effect and mode of action of different nucleotides on the catalytic activity of some eukaryotic and prokaryotic IMPDHs.

	Species (IMPDH acronym)	Ligands	Effect and mode of regulation	References
Eukaryotes	*Ashbya gossypii* (IMPDHag)	XMP	Competitive inhibitor for IMP	Buey et al. (2015a)
		GMP	Competitive inhibitor for IMP	Buey et al. (2015b)
		GDP	Allosteric inhibitor	Buey et al. (2015b)
		GTP		
		Ap4A	Allosteric activator	Fernández-Justel et al. (2019)
		Ap5A		
		Ap6A		
		Ap5G	Allosteric activator; Increase sensitivity to GTP/GDP mediated inhibition	Fernández-Justel et al. (2019)
	*Cryptosporidium parvum*	GMP	Competitive inhibitor for IMP	Umejiego et al. (2004)
	*Homo sapiens* type I (hIMPDH1)	XMP	Competitive inhibitor for IMP	Carr et al. (1993)
		GMP		
		GDP	Allosteric inhibitor	Buey et al. (2015b)
		GTP		
		Ap5A	Allosteric activator that reverts the inhibition by GDP	Fernández-Justel et al. (2019)
		Ap5G	Allosteric inhibitor that increases sensitivity to GTP/GDP mediated inhibition	Fernández-Justel et al. (2019)
	*Homo sapiens* type II (hIMPDH2)	XMP	Competitive inhibitor for IMP	Carr et al. (1993)
		GMP		
		GDP	Allosteric inhibitor	Buey et al. (2015b)
		GTP		
	*Leishmania donovani*	XMP	Competitive inhibitor for IMP	Dobie et al. (2007)
		GMP	Noncompetitive inhibition	Dobie et al. (2007)
		GTP		
	Rat	XMP	Competitive inhibitor for IMP	Jackson et al. (1977)
		GMP		
		AMP		
	*Sus scrofa domestica*	XMP	Competitive inhibitor for IMP	Pugh and Skibo (1993)
		GMP		
		AMP		
	*Tritrichomonas fœtus*	XMP	Competitive inhibitor for IMP	Verham et al. (1987)
		GMP		
	*Trypanosoma brucei* (IMPDHtb)	GMP	Allosteric ligand	Nass et al. (2020)
		ATP		
Prokaryotes	*Aerobacter aerogenes*	XMP	Competitive inhibitor for IMP	Brox and Hampton (1968), Heyde et al. (1976)
		GMP		
	*Bacillus anthracis*	XMP	Competitive inhibitor for IMP	Makowska-Grzyska et al. (2012)
	*Bacillus subtilis* (IMPDHbs)	XMP	Competitive inhibitor for IMP	Wu and Scrimgeour (1973), Ota et al. (2008)
		GMP		
		AMP	Mix of competitive and noncompetitive inhibition for IMP	Ota et al. (2008)
		ADP		
		ATP		
		Ap4A	Allosteric inhibitor	Giammarinaro et al. (2022)
		(p)ppGpp	Allosteric inhibitor only in the presence of ATP	Fernandez-Justel et al. (2022)
	*Borrelia burgdorferi*	XMP	Competitive inhibitor for IMP	Zhou et al. (1997)
		GMP		
	*Campylobacter jejuni* (IMPDHcj)	GMP	Competitive inhibitor for IMP	Yu et al. (2019)
	*Escherichia coli* (IMPDHec)	XMP	Competitive inhibitor for IMP	Powell et al. (1969), Kerr and Hedstrom (1997)
		GMP	Competitive inhibitor for IMP	Gilbert et al. (1979)
		GTP	Allosteric inhibitor only in the presence of ATP	Fernandez-Justel et al. (2022)
		ppGpp ppGp	Competitive inhibitor for IMP	Pao and Dyess (1981)
	*Legionella pneumophila*	ATP	Allosteric activator	Alexandre et al. (2015)
	*Mycobacterium tuberculosis* (GuaB2)	XMP	Inhibitor	Usha et al. (2011)
	*Neisseria meningitidis*	ATP	Allosteric activator	Alexandre et al. (2015)
	*Pseudomonas aeruginosa* (IMPDHpa)	ATP	Allosteric activator	Labesse et al. (2013)
		GTP	Allosteric inhibitor only in the presence of ATP	Fernandez-Justel et al. (2022)
		Ap4A	Allosteric activator	Despotovic et al. (2017)
	*Streptomyces coelicolor* (IMPDHsc)	(p)ppGpp	Allosteric inhibitor only in the presence of ATP	Fernández-Justel et al. (2022)

Finally, the synthesis of the central precursor of the DNPNB pathway, namely, PRPP (Hove-Jensen et al., 2017), is also tightly regulated. PRPP synthetase catalyzes PRPP formation using R_5_P and ATP as substrates. This enzyme is inhibited by purine nucleotides (Gibson et al., 1982; Becker and Kim, 1987; Nosal et al., 1993; Eriksen et al., 2000). In particular, a complex inhibitory pattern by ADP has been characterized being either a competitive or an allosteric inhibitor depending on the R_5_P concentration (Switzer and Sogin, 1973; Hove-Jensen et al., 1986; Hove-Jensen et al., 2017).

## 3 The multiple facets of allosteric regulation in IMPDH

IMPDH (step 11, Figure 2) belongs to the oxidoreductase family of enzymes, which catalyzes the NAD^+^-dependent oxidation of IMP to XMP. Despite the lack of thermodynamic studies of the chemical reaction in question, the IMPDH-catalyzed reaction is historically described as one of the DNPNB rate-limiting steps (Hedstrom, 2009).

Humans and other mammals have two genes encoding type 1 and type 2 IMPDHs (denoted hIMPDH1 and hIMPDH2, respectively). The two human isoforms (84% identity) are expressed to varying extents in most tissues. However, some exceptions exist, such as in the case of retina where hIMPDH1 is predominant (Hedstrom, 2009). On the other hand, most bacteria have only one gene (named *guaB*) encoding IMPDH. An exception arises in the case of the bacterium *Mycobacterium tuberculosis*, which possesses three genes (*guaB1*, *guaB2* and *guaB3*) predicted to code for IMPDH: however, only one of them (*guaB2*) has been demonstrated to actively encode an IMPDH (Usha et al., 2011).

### 3.1 Catalytic and structural properties of IMPDHs

**FIGURE 6:**
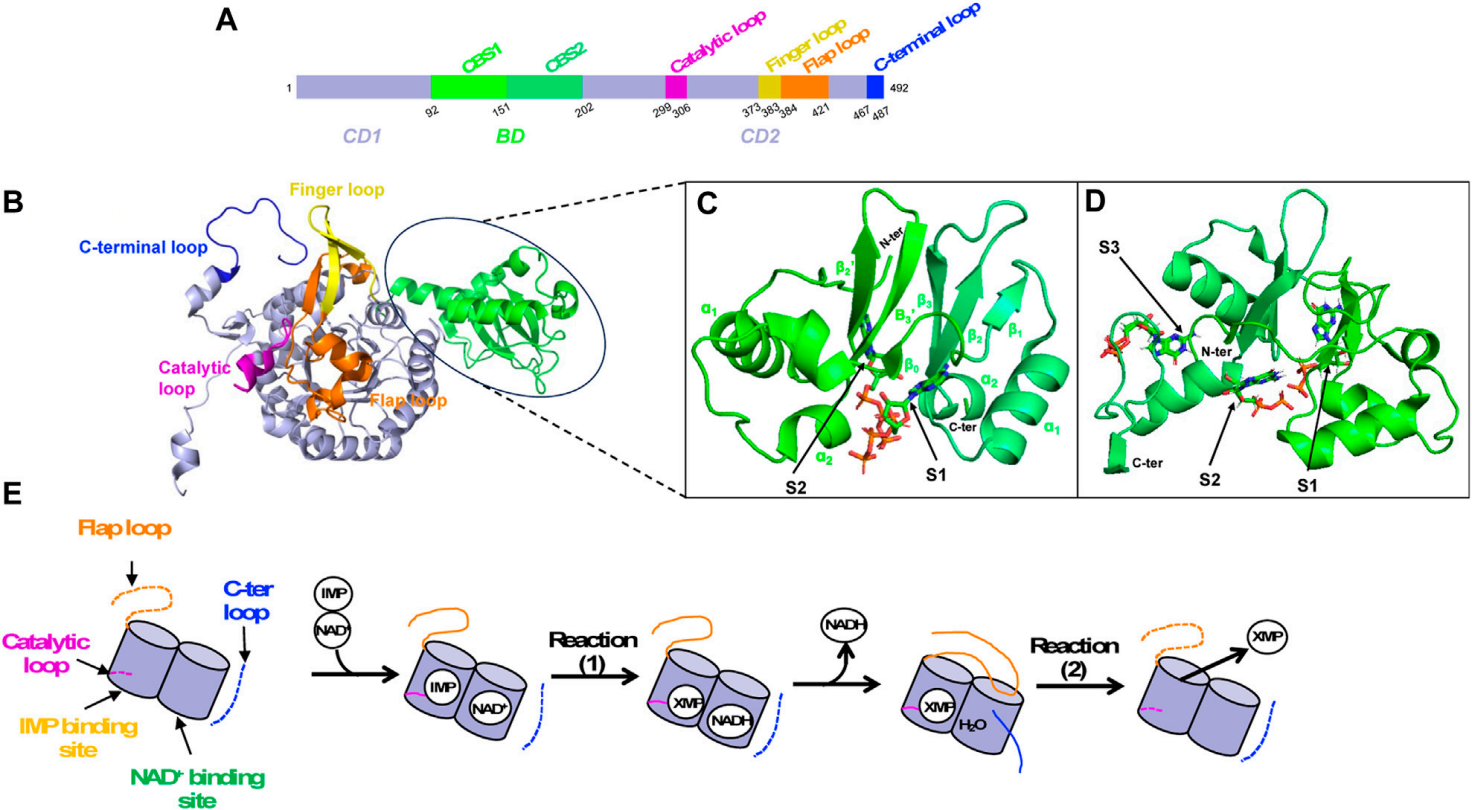
IMPDH structural overview. **(A)** Primary sequence as a schematic bar representation to position the Bateman domain (BD, green) composed of two CBS modules and the catalytic domain (CD, lavender). Important loops of the catalytic domain are colored as follows: catalytic loop in pink, finger loop in yellow, flap loop in orange and C-terminal loop in blue. **(B)** 3D structure of IMPDH monomer in ribbon representation, showing the Bateman domain and the catalytic domain with important loops (same color as in A). **(C, D)** Zoom on the Bateman domain of IMPDHpa (C; PDB 4DQW) and that of IMPDHag (D; PDB 4Z87) with two ATP molecules and three GDP molecules shown in sticks, respectively. **(E)** Summary diagram of the structural transitions at the level of the loops of the catalytic domain (same color code as in A) during catalysis. When loops are not ordered, they are represented as dashed lines.

The monomeric canonical form of IMPDH consists of 400–550 amino acids. It is made up of two structural domains, the catalytic domain and the so-called Bateman domain. This second domain (denoted BD) is nested in the catalytic domain at the primary sequence level (Figure 6A), thus dividing it into two parts (denoted CD1 and CD2). Even though the Bateman domain is not essential for catalytic function, only few IMPDHs lack it (McMillan et al., 2000; Macpherson et al., 2010).

The catalytic domain is made up of a “β-strand-α-helix -β-strand-α-helix” motif repeated four times forming a (ß/α)_8_ triose-phosphate isomerase (TIM) barrel (Banner et al., 1976). It involves several residues that are critical for substrate binding and catalysis, including the catalytic cysteine completely conserved, an aspartate-tyrosine catalytic dyad where the arginine serves as a general base catalyst and a histidine that helps to stabilize the transition state. Several structural components are crucial for catalysis (Figures 6A, B–D). These include a catalytic loop, which contains the catalytic cysteine, and a finger loop, that also plays a vital role in stabilizing the octameric organization. Another element is the flap loop that facilitates the entry and exit dynamics at the active site, which are essential for catalysis. Lastly, there is a C-terminal loop that contains the arginine/tyrosine dyad important for activating a water molecule involved in the catalytic mechanism.

The Bateman domain is made up of two repeated sequences known as CBS modules. It is named after Geoffrey L. Bateman, a British biochemist who first discovered the domain in cystathionine-β-synthase (Bateman, 1997). Since then, the Bateman domain has been found in many other proteins, and its role in nucleotide binding and regulation is now recognized as an important feature of many metabolic and signaling pathways (Ereño-Orbea et al., 2013; Anashkin et al., 2017). In IMPDH, each CBS module presents the βαββα core, but lacks the upstream α_0_ helix and β_0_ strand (Figure 6C). The role of the Bateman domain in IMPDH has long been a source of controversy. Many works, published later than those characterizing the catalytic domain, have shown an important physiological role of the Bateman domain, especially in hIMPDH1 and in *E. coli* IMPDH (IMPDHec).

In the case of hIMPDH1, two point mutations, R224P and D226N, affecting two highly conserved residues of the second CBS module of the Bateman domain, have been independently identified in individuals with retinal photoreceptor degeneration from a Spanish family and from American families. Other less abundant point mutations (such as T116M and N198K) in the Bateman domain have also been described (Bowne et al., 2002; Kennan et al., 2002; Wada et al., 2005). More recently, retinitis pigmentosa mutations in canonical hIMPDH1 have been divided into two classes based on their sensitivity towards GTP regulation. Class I mutations that regroup five mutations in which hIMPDH1 is insensitive to GTP and class II mutations of hIMPDH1, including four disease mutants, that are inhibited by GTP (Burrell et al., 2022). Since the transduction of light signals in the retina is largely dependent on signaling pathways controlled by G protein-coupled receptors (GPCRs), and therefore depending on GTP concentration, a mutation in IMPDH altering the pool of guanine nucleotides could explain these retinopathies at the cellular level. Nevertheless, the R224P and D226N mutants present a protein folding anomaly, thus altering the oligomeric state of the enzyme while remaining active (Wang et al., 2011). These mutated forms of hIMPDH1 have also been described as being able to form IMPDH filaments (Ji et al., 2006; Labesse et al., 2013; Anthony et al., 2017). More recently, mutations located in the Bateman domain of hIMPDH2 have been identified in patients with neurodevelopmental diseases (Zech et al., 2020).

In *E. coli*, it has been demonstrated that the Bateman domain of IMPDHec plays a role in the regulation of nucleotides (Pimkin and Markham, 2008; Pimkin et al., 2009). A mutant strain, obtained by replacing the sequence encoding the Bateman domain by a *scar* sequence of 24 amino acids, had a similar bacterial growth as the wild-type strain in several rich culture media. On the other hand, it showed a reduced proliferation rate in minimal medium with glucose and adenosine as the sole carbon and nitrogen sources, respectively. Adenosine toxicity has been related to accumulation of cytosolic IMP, given the possibility of conversion of adenosine, but not 2′-deoxyadenosine, to inosine by adenosine deaminase. This mutation also induced a disturbance of the pool of nucleotides compared to the wild-type strain, illustrated by an increase to non-physiological concentrations of ATP and by a slight decrease in the concentration of GTP. In addition, a decrease in the enzymatic activity of this mutant has been observed in the lysate in comparison with the wild-type form due to the instability of the protein (Pimkin et al., 2009). Thus, all together, these results are in favor of a link between the deletion of the Bateman domain in IMPDHec and the increase in the cellular concentration of IMP, which can activate the synthesis of adenine nucleotides and inhibit the synthesis of guanine nucleotides.

The role of the Bateman domain was further characterized at the molecular and physiological level by studying wild-type and mutant IMPDHs from different species. This led to the classification of IMPDHs into two classes (Alexandre et al., 2015). Class I and class II IMPDHs exhibit different catalytic properties and structural features and are thought to have evolved from different ancestral enzymes. The common effector of both classes is MgATP, which binds into the Bateman domain but leads to dissimilar properties. Class I IMPDHs are found in three Gram-negative bacteria and are typically octameric in solution that are activated by MgATP. These enzymes also exhibit positive homotropic cooperativity towards IMP, but not towards NAD^+^. In contrast, class II IMPDHs are tetramers in the apo form, that switch into octamers in the presence of saturating concentrations of MgATP that has no effect on their catalytic activity. They behave as Michaelis-Menten enzymes for both substrates. This class includes orthologs from both Gram-positive and Gram-negative bacteria, it also regroups IMPDH from eukaryotic organisms such as hIMPDH1 and hIMPDH2 isoforms (Carr et al., 1993; Nimmesgern et al., 1999; Labesse et al., 2013), and the IMPDH of the fungus *Ashbya gossypii* [IMPDHag; (Buey et al., 2017)]. Interestingly, the interchange of the Bateman domain between representatives of both classes leads to the transplantation of the allosteric kinetic regulation or quaternary structure modulation. These recent results demonstrate the key role of the Bateman domain on the IMPDH molecular behaviors and confirm its implication in *E. coli* physiology (Gedeon et al., 2023a).

### 3.2 A multifaceted approach to modulating IMPDH activity and quaternary structure

IMPDHs are mostly known to be regulated by feedback inhibition through end-product nucleotides and can be allosterically modulated by nucleotides and di-nucleoside phosphates. A summary of these different regulations of IMPDHs from various species is given in Table 2.

The vast majority of IMPDHs from prokaryotes or eukaryotes are regulated by feedback inhibition either by XMP or GMP. The first example came from the first isolated IMPDH (Magasanik et al., 1957) from the bacterium *Aerobacter aerogenes* (renamed *Klebsiella aerogenes*). In the case of IMPDHag, SAXS experiments showed a more compact tetramer in the presence of GMP than that in the apo condition, illustrated by the decrease in the radius of gyration of 1 Å (Buey et al., 2015b).

IMPDH allosteric regulation is also a highly conserved mechanism among various species. ATP has been the first nucleotide described as an allosteric regulator of IMPDHs. It acts either on the kinetic parameters (class I) or on the oligomeric state (class II) (Labesse et al., 2013; Alexandre et al., 2015; Labesse et al., 2015). In the case of class I IMPDHs, full activation by ATP needs the presence of a divalent cation such as Mg^2+^ or Mn^2+^. Two binding sites within the two canonical binding sites in the Bateman domain [referred to as S1 and S2 (Ereño-Orbea et al., 2013)] have been identified in the crystal and cryoEM structures of IMPDHpa (Labesse et al., 2013). Comparison of these structures with the apo one has revealed modifications of the global shape of the IMPDHpa octamer upon MgATP binding, the apo one being much more compact than the one in complex with MgATP (Alexandre et al., 2019). Furthermore, crucial loops (Figure 6) involved in the catalytic mechanism are ordered in the apo form but are not visible in the activated form and thus supposed to be flexible, which would explain the increased catalytic efficiency of the enzyme in the presence of MgATP.

Different guanine nucleotides have also been described as IMPDH effectors (see Table 2). End-product nucleotides, such as GDP and GTP, act as allosteric inhibitors by binding to the Bateman domain. This mode of regulation was first described for IMPDHag as a model and was considered at that time to be specific for eukaryotic IMPDHs (Buey et al., 2015b). Resolution of the structure of IMPDHag in the presence of GDP revealed the presence of three molecules of GDP per monomer. Two GDPs bind to the Bateman domain in the two canonical sites S1 and S2. The third GDP molecule binds at a third site (denoted S3) specific for eukaryotic IMPDHs and formed of residues of the α_1_ and α_2_ helices but also of a loop of the catalytic domain. The effects of GDP and GTP on the enzymatic activity of hIMPDH1 and hIMPDH2 are similar to those observed for IMPDHag. On the other hand, guanine nucleotides alone are not effective at inhibiting the catalytic activity of some bacterial IMPDHs (Buey et al., 2015b). Only when ATP is present can GTP or GDP inhibit IMPDHec or IMPDHpa (Fernandez-Justel et al., 2022). This suggests that the relative ratio of ATP and GTP pools is involved in the modulation of the catalytic activity of proteobacterial IMPDHs (Fernández-Justel et al., 2022).

Structural studies have been performed on different IMPDHs in complex with various guanine and adenine nucleotides or mixtures to get a better insight at the molecular level of the impact of their binding to the Bateman domain. SAXS experiments with different nucleotide concentrations showed that it is not only GDP and GTP, but also AMP, ADP and ATP, which induce a switch of IMPDHag from a tetramer to an octamer (Buey et al., 2017). In addition, adenine nucleotides act at lower concentrations than guanine nucleotides. These observations of the modification of the quaternary structure but not of the kinetic parameters by ATP show a molecular behavior of IMPDHag identical to that of class II bacterial IMPDHs (Alexandre et al., 2015). Resolution of the crystal structure of IMPDHag in the presence of ATP (PDB 5MCP), or a mixture of ATP and GDP (PDB 5TC3), led to the description of conformational changes of octamers by these two effectors (Buey et al., 2017). In the structure in the presence of ATP, two molecules per Bateman domain at the two canonical sites S1 and S2 were observed, as previously depicted for IMPDHpa (Labesse et al., 2013). Moreover, an elongated structure is observed with an approximate dimension of 115 Å. In the presence of a mixture of ATP and GDP, the former binds in the S1 site while the latter binds at the S2 site and its specific S3 site (Buey et al., 2017). Under these conditions, IMPDHag is in the form of a compact octamer (dimension 90 Å), identical to the octamer in the presence of GDP alone. The same behaviors (octamer compaction and finger interactions) have been observed for IMPDHpa inhibitory forms (Labesse et al., 2013; Alexandre et al., 2019) and human IMPDHs (Anthony et al., 2017; Burrell et al., 2022).

More recently, by overexpressing IMPDH from the parasite *Trypanosoma brucei* (denoted IMPDHtb) with the aim of purifying the enzyme after transfection with a baculovirus containing a plasmid encoding IMPDHtb in Sf9 insect cells, in-cell crystals of the enzyme were observed (Nass et al., 2020). The resolution of the structure of this enzyme led to the identification of an ATP molecule in the S1 site and, surprisingly, of a GMP molecule at the S2 site of the Bateman domain (described as a competitive IMP inhibitor for most IMPDHs and never as an allosteric ligand). In addition, this structure organizes into an octamer, with a conformation similar to that of IMPDHag in the presence of GDP, or the mixture of ATP and GDP. However, the effect of these two nucleotides on IMPDHtb kinetics remains to be tested.

Besides allosteric regulation by downstream nucleotides, bacterial IMPDHs are also regulated by the alarmone (p)ppGpp, which plays a crucial role in the bacterial stress response (Steinchen et al., 2020; Anderson et al., 2021). Recent studies (Fernandez-Justel et al., 2022) have shown that (p)ppGpp can bind to and inhibit IMPDH activity in certain bacterial species, such as *B. subtilis* (IMPDHbs) or *Streptomyces coelicolor* (IMPDHsc). The (p)ppGpp binding site in IMPDH overlaps with the second canonical site, allowing direct competition between (p)ppGpp and ATP to modulate catalytic activity. The mechanism of inhibition involves the binding of (p)ppGpp to the Bateman domain, in the presence of ATP, resulting in a conformational change that reduces IMPDH catalytic activity. Resolution of the structure of IMPDHsc in the presence of (p)ppGpp showed the formation of octamers assembled with a conformation that is similar to that of IMPDHpa in the presence of ATP and GDP. On the other hand, the same authors (Fernandez-Justel et al., 2022) reported that (p)ppGpp had no detectable effect on the activity of IMPDHec and IMPDHpa, regardless of the presence or absence of ATP. Surprisingly, in the case of IMPDHec, another team has been demonstrated that not only ppGp but also ppGpp are competitive inhibitors for IMP with Ki values of 22 and 48 μM, respectively (Pao and Dyess, 1981).

Eukaryotic and prokaryotic IMPDHs are also modulated by some di-nucleoside phosphates such as Ap4A, Ap5A, Ap5G and Ap6A, which bind to the Bateman domain. Opposite effects have been described for Ap4A (Despotovic et al., 2017; Fernández-Justel et al., 2019). In all reported cases, it competes for ATP, although with lower affinities: hence, the physiological significance of Ap4A binding is being called into question. In the case of IMPDHec, no impact is observed on the catalytic activity, whereas it is an allosteric activator for IMPDHpa and IMPDHag. On the other hand, Ap4A is an allosteric inhibitor of IMPDHbs. It binds at the interface between two IMPDH subunits inducing a switch from tetramers into octamers (Giammarinaro et al., 2022). Ap5A and Ap6A can also compete for adenine/guanine nucleotides for the canonical sites (Fernández-Justel et al., 2019). They are allosteric activators for IMPDHag. They also compete for GDP, inducing a conformational switch to an extended form that reverts the strong IMPDHag inhibition by GDP. The same behavior is observed with Ap5G being an allosteric activator. However, Ap5G potentializes the IMPDHag sensitivity to GTP/GDP-mediated allosteric inhibition by inducing the formation of compacted-inhibited octamers. In the case of other eukaryotic IMPDHs, and more specifically of hIMPDH1, Ap5A and Ap6A showed no impact on the hIMPDH1 catalytic activity. On the other hand, Ap5A but not Ap6A reversed the GDP-induced inhibition. In addition, Ap5G can hypersensitize hIMPDH1 to GTP/GDP-mediated allosteric inhibition as found for IMPDHag.

Briefly, all of these results show a synergistic regulation between adenine and guanine nucleotides for IMPDHs, with in particular inter-species variations in the specificity of effector binding in the Bateman domain.

In addition to the modulation of IMPDH enzymatic activity, nucleotides could also impact their oligomeric states. It has been described for class II IMPDHs that ATP promotes the formation of octameric species (Alexandre et al., 2015). In eukaryotes, IMPDH has been shown to form filaments or cytoophidia (Guo and Liu, 2023): in zebrafish (Keppeke et al., 2020) and in humans (Labesse et al., 2013; Keppeke et al., 2015; Anthony et al., 2017; Chang et al., 2018; Fernandez-Justel et al., 2019; Chang et al., 2022). As summarized in a recent review (Buey et al., 2022), the formation of IMPDH filaments is regulated by various factors, including nucleotide binding, such as ATP/GTP (Anthony et al., 2017; Keppeke et al., 2018; Fernandez-Justel et al., 2019; Johnson and Kollman, 2020; Burrell et al., 2022), post-translational modifications (Plana-Bonamaisó et al., 2020), and interactions with other proteins in particular with CTP synthase (Keppeke et al., 2015; Chang et al., 2018; Hayward et al., 2019). It has been demonstrated that IMPDH cytoophidia and filaments are implicated in various cellular processes including cell cycle regulation and nucleotide homeostasis. Moreover, aberrant filament formation or altered dynamics have been linked to retinopathy-associated mutations (Fernandez-Justel et al., 2019; Burrell et al., 2022) and other diseases, including cancer and neurodegenerative disorders (Calise and Chan, 2020; Keppeke et al., 2020; Ruan et al., 2020; Burrell and Kollman, 2022).

## 4 Navigating the complexities of metabolon formation for finer regulation of DNPNB

As the discovery of the diverse modes of enzymatic activity modulation rose during the years, it was clear that the control of metabolic fluxes was a result of not only metabolite-mediated regulations, but also physical protein-protein interactions that lead to the formation of megacomplexes. The search for the first evidence of the existence of such regulation was initiated by the group of David E. Green while studying the enzymes of the Krebs cycle in eukaryotic cells (Green et al., 1948) and then thoroughly conducted by the work of Paul A. Srere and colleagues (Srere, 1972; Srere et al., 1973; Halper and Srere, 1977; Srere, 1987; Vélot et al., 1997). The ensemble of these papers was in favor of a central position of three of the enzymes (malate dehydrogenase, citrate synthase and aconitase) in a Krebs cycle enzymic megacomplex implicating all enzymes of the pathway, and that substrate channeling existed between several of the enzymes. In 1985, Srere subsequently termed and defined the “metabolon” as a functional supramolecular complex that regroups sequential enzymes of a particular metabolic pathway to favor the channeling of metabolites between active sites (Srere, 1985). Metabolons are characterized by the absence of membranes surrounding the enzymes, but can be tightly associated to the cytoskeleton, cell membranes, or intracellular domains of membrane proteins. Enzymes within a metabolon are transiently maintained by weak interactions that can be formed or disrupted by variations in metabolite concentrations or cellular signals (Ginsburg and Stadtman, 1970). Since then, with the development of novel techniques such as two hybrid systems, proteomics, fluorescence microscopy, microfluidics, and structural and computational biology, proofs for the existence of a Krebs cycle metabolon were added (Wu et al., 2014; Wu and Minteer, 2015), and the identification and characterization of several other metabolons in eukaryotes and prokaryotes have been possible (Kastritis and Gavin, 2018; Zhang and Fernie, 2021).

### 4.1 The purinosome, a mammalian purine biosynthesis metabolon

**FIGURE 7:**
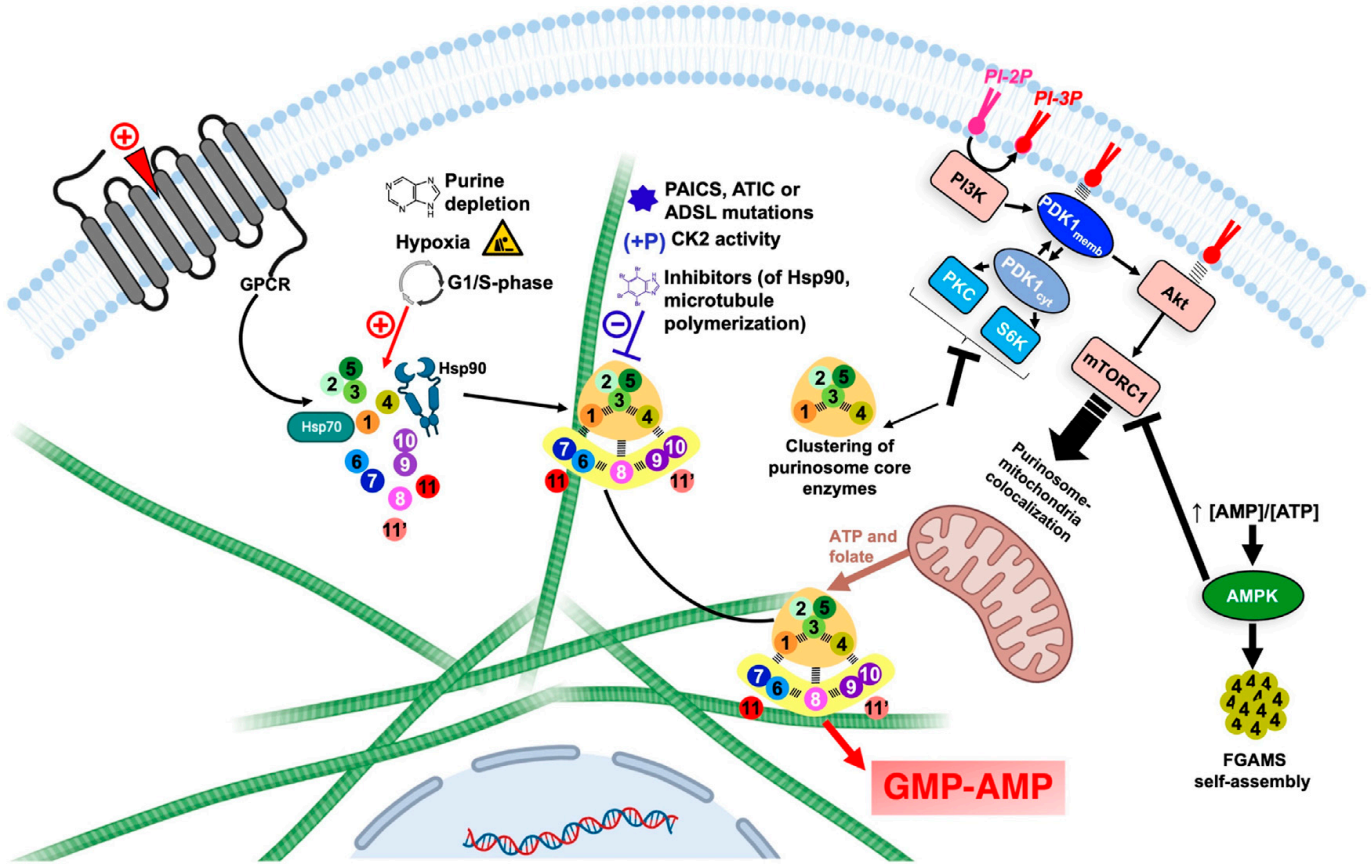
Overview of the regulation of DNPNB enzyme clustering and purinosome assembly-disassembly under the control of regulatory proteins and signaling pathways. Each circle corresponds to one step of the DNPNB pathway: same color code and numbering as in Table 1. The green filaments represent microtubules. The figure was created with Biorender.com. AMPK, AMP-activated protein kinase; CK2, casein kinase 2; PDK1, 3-phosphoinositide-dependent kinase-1; PI3K, phosphatidylinositol 3-kinase; PI-2P, phosphatidylinositol (4,5)-bisphosphate; PI-3P, phosphatidylinositol (3,4,5)-trisphosphate; PKC, protein kinase C; S6K, p70 ribosomal S6 kinase.

While proposing the term “metabolon,” Srere cited several metabolic pathways for which a metabolon could potentially exist, including the biosynthesis of purine nucleotides, which he named “purinogenic metabolon” (Srere, 1985). Intriguingly, after the description of purine nucleotide biosynthetic enzymes from extracts of pigeon liver by Hartman and Buchanan in 1959 (Hartman and Buchanan, 1959), speculations of the existence of such an assembly were based on experiments of co-purification of several of the enzymes from pigeon liver (Rowe et al., 1978) and human lymphocytes (McCairns et al., 1983), but this only in the presence of polyethylene glycol. Furthermore, the application of low-intensity sonication have shown to induce a reversible arrest of nucleotide biosynthesis in yeast, with reactivation upon cessation of sonication (Burns, 1964). In 2001, Benkovic’s team firstly attempted to validate the existence of such organization in mammalian cells in a rich fetal bovine serum medium culture (Gooljarsingh et al., 2001). Nonetheless, in 2008, the pioneer work of the same team led to the identification of a complex specialized in the biosynthesis of purine nucleotides in mammals, baptized “purinosome” (An et al., 2008). In this work, HeLa cells were grown in purine-depleted medium and co-transfected with one construct encoding FGAMS (step 4) fused to orange fluorescent protein and one of the *de novo* biosynthetic enzymes PPAT (step 1), TrifGART (steps 2,3 and 5), PAICS (steps 6,7) ADSL (steps 8,12′) or ATIC (steps 9, 10) fused with green fluorescent protein. In these new culture conditions, cytoplasmic clusters and colocalizations were observed. These cellular structures were also shown to be reversed when medium culture was supplemented with purines. Since then, many properties of this megacomplex (succinctly summarized in Figure 7 and reviewed below and elsewhere (Pedley and Benkovic, 2017; Pareek et al., 2020a) have been minutely detailed by Benkovic’s team and other groups.

#### 4.1.1 Substrate channeling and subcellular localization

From a functional point of view, the formation of the purinosome was correlated with an increase in the DNPNB rate by measurements of radioactivity incorporation in HeLa cells in the presence of ^14^C-glycine (An et al., 2010a). Nucleotide quantification by liquid chromatography coupled to tandem mass spectrometry also validated these results and revealed that purinosome formation is accompanied with a 1.2–1.5-fold increase in mono/di/tri-phosphate purine nucleotides concentration and 3-fold increase in IMP concentration, the substrate of IMPDH and GMPS at the branching point towards GMP and AMP synthesis (Zhao et al., 2015). Recently, state-of-art techniques employing metabolomic, mass spectrometry imaging and mathematical simulation were also employed to directly detect channeling between DNPNB enzymes (Pareek et al., 2020b). In this work, the metabolic flux of DNPNB in cells incubated with ^13^C,^15^N-serine showed AMP and GMP proportions that are different from theoretical values estimated if the enzymes of the pathway were considered to be diffused in the cytoplasm. Additionally, *in situ* imaging of unique cells by mass spectrometry revealed regions that could be occupied by active purinosomes that are proximal to mitochondria. These regions were also shown to be highly concentrated in the DNPNB intermediate AICAR (substrate of AICART, step 9) and where ATP synthesis takes place.

Other investigations also led to the conclusion that the purinosome is not an isolated complex but in direct interaction with the cytoskeleton. Indeed, FGAMS, considered as a marker of purinosomes, was shown to colocalize with microtubule filaments but not actin filaments. Subsequently, addition of nocodazole, but not cytochalasin D, was shown to alter purinosome formation and to decrease 38% purine nucleotides concentration (An et al., 2010a). The association to the microtubule filaments also favors the translocation of the purinosome close to the mitochondria, as noted in fibroblasts lacking the hypoxanthine-guanine phosophoribosyltransferase, and in HeLa cells in purine-depleted conditions (French et al., 2016; Chan et al., 2018). This physical proximity between purinosome and mitochondria is mutualistic, with the first benefiting from free ATP and folic cofactors, and the second being able to directly recycle ADP into ATP after translocation *via* an ADP/ATP transporter at the surface of mitochondria (Tibbetts and Appling, 2010; Kunji et al., 2016; Pedley and Benkovic, 2017).

#### 4.1.2 Regulation of purinosome assembly-disassembly in cells

Temporal modulation of purinosome formation depends on cellular needs in nucleotides. Indeed, during cellular proliferation, *de novo* purine biosynthesis is most highly active during phases where DNA replication takes place, mainly from mid-G1 phase until the end of the S phase (Fridman et al., 2013). This activity was shown to correlate with a 3.8-fold increase of the number of purinosomes per cell during these two steps, followed by a drastic decrease in purinosomes at the end of G2 phase when DNA synthesis is accomplished and cells are ready to divide (Chan et al., 2015).

Purinosome assembly can also possibly be modulated by environmental factors. In fact, hypoxia was shown to induce the formation of purinosome, but strangely without a boost in DNPNB (Doigneaux et al., 2020).

Several regulations on a cellular level were also shown to take place *via* regulatory proteins and signaling pathways (Figure 7). For example, pro-mitotic signals induced by agonists of purinergic and α_2A_ GPCR regulate the formation of purinosome (Verrier et al., 2011; Fang et al., 2013). Additionally, several kinases were shown to be involved in DNPNB regulation and metabolon formation. AMP-activated protein kinase (AMPK) for example, can negatively regulate the formation of the megacomplex. This energy-sensing enzyme is activated after an increase of AMP/ATP ratios in cell and is therefore able to regulate sugar, lipid and energetic metabolism (Hardie et al., 2006). In the presence of an AMPK activator, FGAMS was shown to be exclusively agglomerated in clusters in the absence of other DNPNB enzymes. This enzyme sequestration is accompanied with a decrease in *de novo* metabolic flux, illustrated by the decrease of 44% and 80% of AMP and GMP pools in cells, respectively (Schmitt et al., 2016). Moreover, casein kinase 2 (CK2) can phosphorylate PPAT, TrifGART and FGAMS, whereas protein kinase B (PKB, also known historically as Akt) is able to phosphorylate FGAMS. Inhibition of CK2 induces the formation of purinosome, however inhibition of all isoforms of Akt (Akt1, Akt2 and Akt3) did not show any effect on purinosome formation, meaning that purinosomes are not directly regulated through an Akt-dependent pathway (An et al., 2010b).

Regulation via 3-phosphoinositide-dependent kinase-1 (PDK1) was also extensively studied. This serine/threonine kinase can translocate between the membrane or the cytosol depending on the phosphorylated state of phosphatidylinositol in the membrane. Indeed, the activation of phosphatidylinositol 3-kinase (PI3K) by several possible agonists prompts it to catalyze the conversion of the phosphatidylinositol (4,5)-bisphosphate (PI-2P) into phosphatidylinositol (3,4,5)-trisphosphate (PI-3P), that is recognized by PDK1 that can therefore translocate to the membrane (Komander et al., 2004). In the cytosolic state, PDK1 activates pathways that depend on several other kinases, such as protein kinase C (PKC) and p70 ribosomal S6 kinase (S6K) (Biondi et al., 2001); conversely, membrane-associated PDK1 activates the Akt-dependent pathway and sequentially the mammalian target of rapamycin complex 1 (mTORC1) (Emmanouilidi and Falasca, 2017). Inhibition of PI3K or Akt showed no effect on purinosome formation, suggesting a non-participation of membrane-associated PDK1-dependent pathways in purinosome regulation; however, inhibition of PDK1, PKC or S6K induced the formation of tripartite clusters solely formed of PPAT, TrifGART and FGAMS, but not other DNPNB enzymes (Sundaram and An, 2015; Schmitt et al., 2018). Surprisingly, inhibition of mTORC1 did not affect the number of purinosomes per cell, but instead, a diminishment of purinosome-mitochondria colocalization (French et al., 2016). mTORC1 is indirectly linked to purinosome cellular localization by activating the expression of *MTHFD2*, a gene encoding a mitochondrial methylenetetrahydrofolate dehydrogenase 2 that ensures release of formate cofactors from inner membrane of mitochondria to the cytosol, therefore securing indispensable cofactors for several DNPNB enzymes (Ben-Sahra et al., 2016). Lately, Ali et al. (2022) validated that mTORC1 enhances DNPNB by increasing the translation of SLC4A7, a specific bicarbonate transporter, and therefore increasing the intracellular concentrations of bicarbonate, crucial for purine and pyrimidine *de novo* biosynthesis. This latter observation illustrates several possible pathways for the indirect implication of mTOR complexes in *de novo* nucleotide synthesis and purinosome regulation.

#### 4.1.3 Purinosome architecture and other protein partners

**FIGURE 8:**
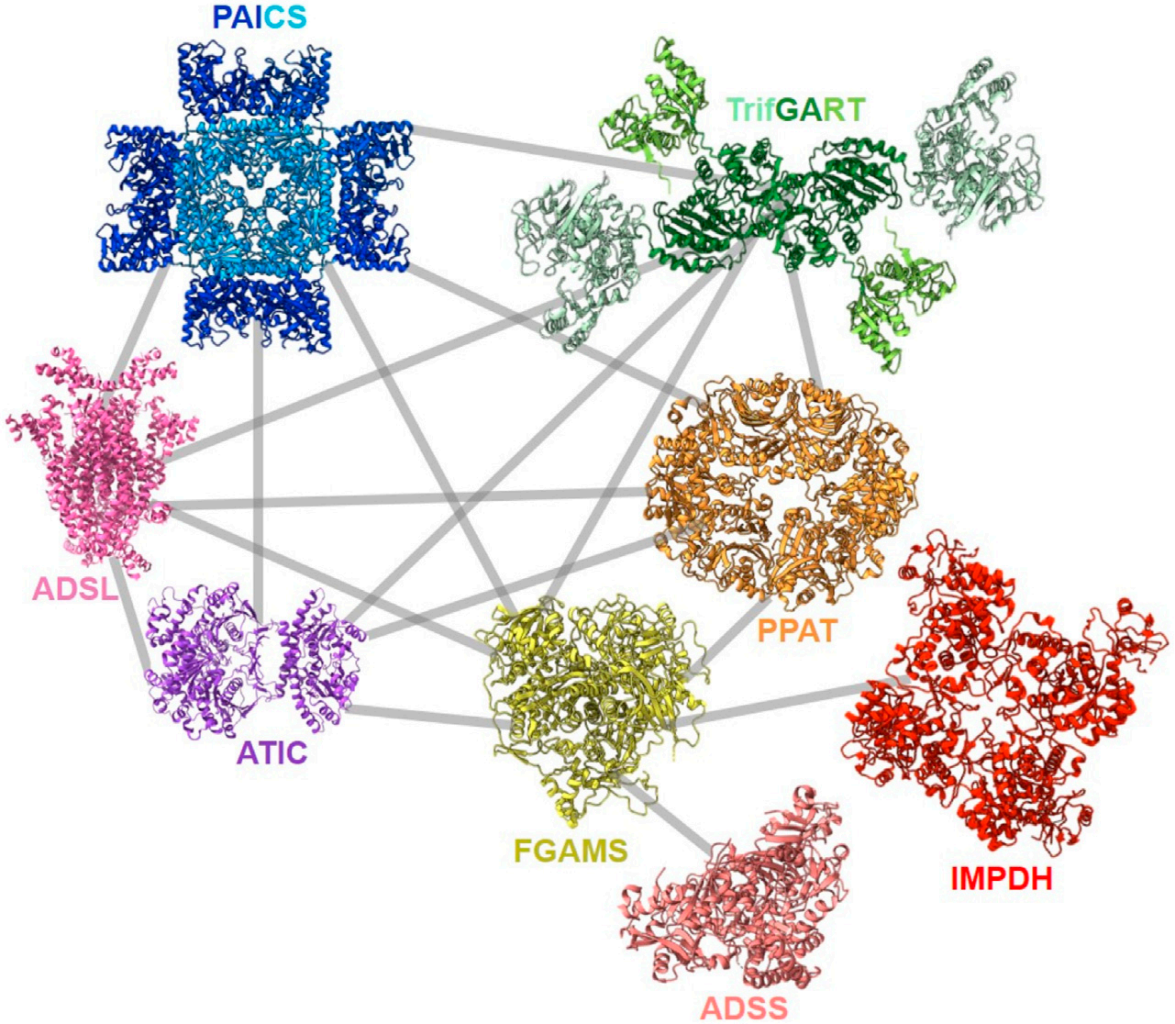
Described protein-protein interactions within the purinosome (Deng et al., 2012; Zhao et al., 2015; Agarwal et al., 2020a; He et al., 2022). Solved 3D-structures of human enzymes (same acronym and color code as in Table 1) have the following PDB accession numbers: 7ALE (PAICS), 4FFX (ADSL), 1PKX (ATIC), 2V40 (hADSS2), 1JCN (hIMPDH1). TrifGART was modeled based on previous published structural data (Welin et al., 2010) and from structures of each of the individual three domains (2QK4 for GARS, 2V9Y for AIRS, 1RBY for GART). The human FGAMS structure was predicted with AlphaFold2 (Jumper et al., 2021). For PPAT, the ortholog from *Arabidopsis thaliana* (PDB 6LBP; 39% identity) is depicted.

After the first evidence of colocalization of DNPNB enzymes, the architecture of the purinosome was studied with the Tango system, a eukaryotic two-hybrid system. With this technique, the binary interaction analysis (Figure 8) of the first six DNPNB enzymes showed a central position of enzymes PPAT, FGAMS, TrifGART and a peripheral position of PAICS, ADSL and ATIC within the purinosome (Deng et al., 2012). Several years later, supplementary fluorescence microscopy experiments revealed that IMPDH and ADSS are also members of the purinosome (Zhao et al., 2015). Moreover, biochemical fractionation of soluble protein extracts followed by tandem mass spectrometry profiling from human cell lines (and eight other phylogenetically distinct eukaryotic species, such as *Drosophila melanogaster*) also led to the identification of a cofractionation of FGAMS, TrifGART, PAICS, ADSL and ATIC into one evolutionarily conserved multiprotein grouping (Wan et al., 2015). Another cofractionation of six proteins including PPAT, hIMPDH1 and hIMPDH2 was also observed using biochemical fractionation on human cell extracts (Havugimana et al., 2012). More recently, a revisited organization of the purinosome was besides proposed, placing PAICS in the center of the megacomplex, being an enzyme capable of interacting with TrifGART, FGAMS, ADSL and ATIC but not PPAT (He et al., 2022). These observations were noted in purine starvation but also in purine rich conditions, meaning that PAICS could potentially play the role of a seeding protein for purinosome formation depending on cellular needs. Very recently, polyubiquitination of PAICS by the ubiquitin ligase ASB11 was shown to favorize the recruitment of the ubiquitin-binding protein UBAP2 and therefore trigger purinosome formation and enhance DNPNB in melanoma cells (Chou et al., 2023). This innovative work sheds lights on the importance of PTMs in mediating purinosome formation (see Section 4) and adds a layer of complexity regarding the regulation of such assemblies.

Other proteins unrelated or indirectly linked to DNPNB were also revealed to be components of the purinosome. Methenyltetrahydrofolate synthetase (MTHFS, EC 6.3.3.2), an enzyme catalyzing the ATP-dependent formation of cofactor 5,10-methenyltetrahydrofolate used by TrifGART and ATIC was shown to colocalize with two tested enzymes of the purinosome, TrifGART and FGAMS (Field et al., 2011). Chaperones and co-chaperones from the Hsp70/Hsp90 system were also validated to regulate the formation of the purinosome (Figure 7) by directly interacting with PPAT and FGAMS (Binder and Pedley, 2013; French et al., 2013; Pedley et al., 2018).

In neural progenitor stem cells and immature neurons from embryo mouse cerebral cortex, the NACHT and WD repeat domain containing protein 1 (Nwd1) of unknown function was also reported as a purinosome participant by interacting with PAICS and FGAMS using yeast two-hybrid and co-immunoprecipitation approaches, respectively (Yamada et al., 2020). Nwd1 was also shown to be essential for purinosome assembly after loss of cluster following knock-down of the gene encoding this protein in neurons. This example proves a possible existence of DNPNB protein partners specific to cell types, and therefore the need to investigate the composition of the purinosome in higher scale of several primary cells and cell lines.

#### 4.1.4 Physio-pathological role of the purinosome

The early studies of mutations in ATIC or ADSL that were linked to neurodegenerative impairments largely contributed to the understanding of the role of the purinosome in diseases.

In the case of ATIC, two mutations were reported in a four-year-old female infant who suffered from AICA-Ribosiduria, a severe congenital neurological disease characterized by an accumulation of dephosphorylated AICAR (Marie et al., 2004). Regarding ADSL, several point mutations have been identified in over 100 cases with varying severity (ranging from fatal neonatal encephalopathy to mild or severe neuronopathic disorders), depending on the ratio of accumulated dephosphorylated SAICAR and AdS (Jaeken and Van den Berghe, 1984; Jaeken et al., 1988; Maaswinkel-Mooij et al., 1997; Kmoch et al., 2000; Mouchegh et al., 2007; Zikanova et al., 2010). Biochemical evaluation of different mutated ADSL revealed no severe alteration of catalytic activity or ability to form the active tetrameric oligomer.

Therefore, to verify if mutations in ADSL and ATIC were able to alter purinosome formation, the group of Zikánová elegantly investigated the megacomplex formation in cultured fibroblasts from the patients presenting the known genetic defects in ATIC or ADSL in purine starvation conditions (Baresova et al., 2012). In the case of the AICA-Ribosiduria patient, the mutated ATIC was not able to form intracellular clusters in the absence of purine. Supplementarily, co-localizations of PPAT-TrifGART and PPAT-ADSL were detected, but to a lesser extent than in the case of wild-type fibroblasts. In the case of ADSL mutation linked to fatal neonatal encephalopathy, no signal for the mutated enzyme was detected in the fibroblasts. However, ADSL was revealed to be in diffused form and/or in clusters from the other clinical cases. Co-localizations of ADSL-ATIC, ADSL-TrifGART and PPAT-ATIC were found to be absent in several of the tested fibroblasts. The ensemble of these observations correlates the mutations to defect in purinosome formation in the case of most of these patients.

More recently, two cases of spontaneous abortion due to a single nucleotide substitution in the gene encoding the bifunctional enzyme PAICS have also been reported. Activity assays revealed a two-fold decrease of the activity of this mutant in comparison with the wild-type counterpart. Furthermore, as the case of ATIC and ADSL mutants, co-localization of PPAT and TrifGART were also absent in fibroblasts from both fetuses (Pelet et al., 2019).

### 4.2 Evidence for the existence of a purinosome equivalent in bacteria

Before the thorough investigation of the purinosome in mammals, studies analyzing three of the DNPNB enzymes in bacteria lead to speculations of a regulation by formation of a prokaryotic metabolon. Rudolph and Stubbe firstly reported kinetic data that support the existence of channeling between purified PurF and PurD of *E. coli* (Rudolph and Stubbe, 1995). Therefore, the existence of a transient association between the first two enzymes of the DNPNB has been hypothesized. Wang et al. (1998) reported the structure of *E. coli* PurD and proposed a docking model to explain the possible channeling of PRA substrate between PurD and PurF. In their model, a channeling complex formed of a PurD monomer and a PurF dimer connected with a funnel between active sites was deduced. However, the search for a stable interaction between PurF and PurD by a plethora of biochemical techniques (gel chromatography, fluorescence spectroscopy, chemical cross-linking, protein affinity chromatography with either PurF or PurD covalently attached to the resin) was unsuccessful. Later-on, structural analysis of small PurL from *B. subtilis* and large PurL from *S. typhimurium* led to the proposal that *E. coli* PurL could be a scaffold for other DNPNB enzymes to form a metabolon (Anand et al., 2004a; Anand et al., 2004b; Hoskins et al., 2004). Despite the development and use of proteomic and genetic techniques to map the protein-protein interaction landscape in *E. coli* ever since (Butland et al., 2005; Arifuzzaman et al., 2006; Hu et al., 2009; Rajagopala et al., 2014), no data has revealed the existence of such interactions between PurL and other DNPNB enzymes, nor between DNPNB enzymes themselves (other than the homo-oligomerization of some DNPNB proteins in some instances).

**FIGURE 9:**
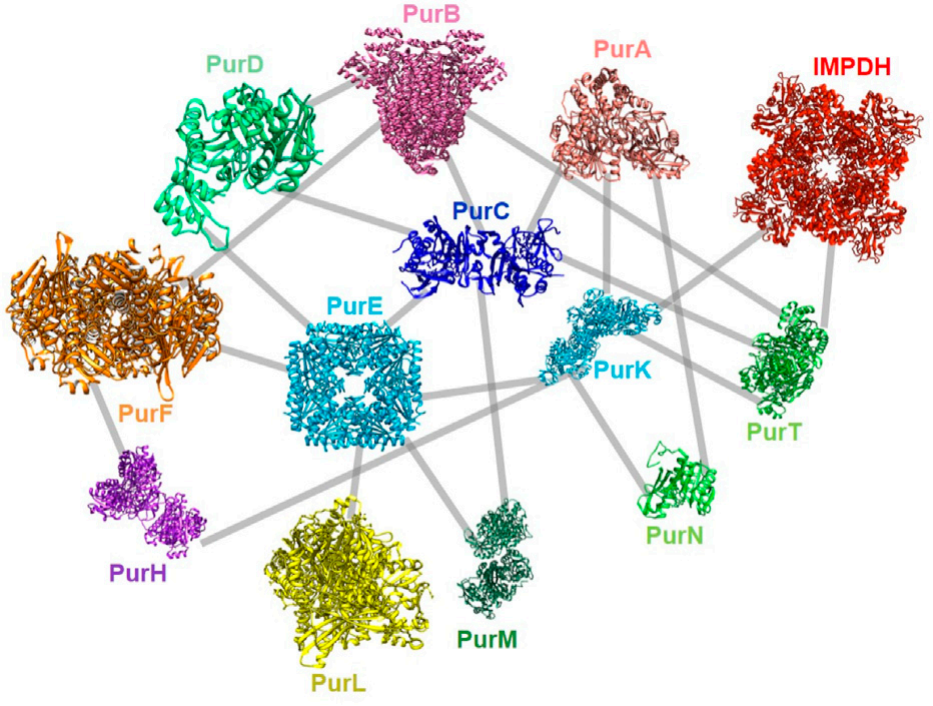
Described protein-protein interactions between *E. coli* DNPNB enzymes (Gedeon et al., 2023b). Each enzyme (same acronym and color code as in Table 1) is represented by its 3D-structure (for the PDB accession numbers, see Figure 2).

Very recently, a global screening of protein-protein interactions between *E. coli* DNPNB enzymes has been performed using a bacterial adenylate cyclase two-hybrid system (Gedeon et al., 2023b). This approach revealed the existence of a dense interaction network between all considered DNPNB enzymes, with PurK, PurE and PurC, being central interactants (Figure 9). PurL was found to be an interactant, but not a scaffold for other DNPNB as proposed previously. The global architecture of these binary interactions seems to be different from early reports of the purinosome, but similar to later results placing PAICS in the center of the purinosome (He et al., 2022). Additionally, alteration of interactions between PurK and some other enzymes of the pathway lead to a decrease in adenylate and guanylate energy charges, meaning that interactions are needed to maintain *ad hoc* concentrations of nucleotides (Gedeon et al., 2023b).

These new observations reinforce the hypothesis of the existence of a regulation via formation of a supramolecular assembly, not only in eukaryotes, but also in bacteria.

### 4.3 Possible clustering of DNPNB enzymes in yeast

In yeast, a systematic screening of metabolic enzymes from yeast GFP strain collection showed that ADE4 (PurF), ADE16,17 (PurH), ADE12 (PurA), three of the IMPDH orthologs (IMD2, IMD3, IMD4) and GUA1 (GuaA) are individually able to form mesoscale intracellular clusters, but not PurDM, PurN, PurE or PurC (Noree et al., 2019), an observation that could raise many supplementary questions on the variations of the architecture of the purinogenic metabolon, if it exists in other species, throughout evolution.

## 5 DNPNB enzymes as promising targets

### 5.1 DNPNB enzymes essentiality in bacteria

Due to its pivotal role in cellular physiology, DNPNB is considered as a highly attractive pathway for the design of novel inhibitors. In human, DNPNB enzymes were linked to multiple neurodegenerative diseases and cancer (Fumagalli et al., 2017; Chen et al., 2022).

In bacteria, several fundamental observations also revealed the importance of DNPNB enzymes in growth, fitness and pathogenicity (Goncheva et al., 2022).

All enzymes of this pathway were found to be essential for *in vitro* bacterial growth of *E. coli* in minimal medium, except for the redundant PurN and PurT enzymes (catalyzing the third chemical step). Additionally, PurB is the only enzyme to be essential not only for growth in minimal medium, but also in rich medium: therefore no *purB* knock-out mutant strain can be generated (Baba et al., 2006). Most of the enzymes were also highlighted as essential for the growth of *E. coli*, *Bacillus anthracis*, *S. typhimurium* and *Staphylococcus aureus* in human serum (Samant et al., 2008; Connolly et al., 2017).

*In vivo* experiments also unveil DNPNB essentiality in virulence and infection. PurA was established as an essential enzyme in pathogenicity of *Streptococcus pneumoniae* (Liu et al., 2021) and *S. aureus* (Lan et al., 2010). PurB, PurL and GuaA were also found to be essential for pathogenicity of *S. aureus* (Mei et al., 1997; Valentino et al., 2014; Connolly et al., 2017). However, some mixed conclusions were highlighted when comparing reported works for other DNPNB genes. Firstly, contrarily to *in vitro* observations, *purN* and *purT* genes were found to be both essential for infection by *S. typhimurium* since *purN* or *purT* mutants were significantly less virulent than wild-type strains in mice (Jelsbak et al., 2016). Secondly*, purK* and/or *purE* seem to be reported as essential or non-essential genes for infections depending on the studied bacterial species and/or the *in vivo* physio pathological context. In *B. anthracis*, the growth in human blood of both *purE* and *purK* mutants is severely impaired. On the other hand, only *purE* mutant showed an attenuated virulence in a murine infection model, and *purK* mutant surprisingly exhibits similar virulence as the wild-type reference strain (Samant et al., 2008). In *Shigella flexneri*, deletion of *purE* showed normal multiplication in HeLa cells (Cersini et al., 1998), whereas in *S. pneumoniae*, *purE*, but also *purK* (additionally to *purL* and *purC*) were identified as virulence genes and deleted mutants showed compromised septicemia in mice (Polissi et al., 1998). In murine models for infection by *Yersinia pestis*, *purK* (and also *purH*) was found to be associated to *in vivo* virulence (Flashner et al., 2004). Deletion of *purEK* operon in *P. aeruginosa* also showed compromised virulence in mice (Wang et al., 1996). A large number of DNPNB genes, of which *purK*, were also linked, when disrupted, to decreased lung persistence in murine pneumoniae model for *Acinetobacter baumannii* (Wang et al., 2014). Surprisingly, bacterial growth of an *E. coli purK* mutant was successfully rescued by complementation with a plasmid overexpressing PurE despite their differential enzymatic activities. These observations were explained by the possibility of favoritism towards a spontaneous conversion of AIR into NCAIR in these conditions independent of PurK, and therefore the rescue of bacterial growth (Firestine et al., 1994; Patrick et al., 2007). Thirdly, depending on the bacteria considered, different conclusions were drawn regarding the essentiality of the *guaB* gene coding for IMPDH, either using deletion mutants or inhibitory compounds. A *S. aureus* mutant strain expressing a catalytically inactive IMPDH showed similar infective potential in comparison with wild-type strain in mice, meaning that targeting of the IMPDH active site is not a good strategy for anti-staphylococcal treatments (Modi et al., 2021). However, using a transposon mutant library, the *S. aureus guaB* gene had been previously found to be essential in infection conditions (Valentino et al., 2014). The essentiality of the *guaB* gene has been also proved for numerous bacterial pathogens (Hedstrom et al., 2011). It includes *Pseudomonas aeruginosa* (Liberati et al., 2006; Lee et al., 2015), *Francisella tularensis* (Santiago et al., 2009), *M. tuberculosis* [only *guaB2* gene, (Sassetti et al., 2001; Sassetti et al., 2003)]. In this latter case, the situation is even more complex as opposite conclusions were obtained from two groups while developing new drugs against *M. tuberculosis*: in one case, *M. tuberculosis* IMPDH was validated as a promising drug target in mouse infection model (Singh et al., 2017), whereas in the other case it was qualified as “not vulnerable” in an *in vivo* context (Park et al., 2017).

The ensemble of all these distinct observations illustrates possible variations depending on the pathogenic potential or molecular niches. Still, it is undeniable that DNPNB enzymes are important for bacterial physiology, and several efforts were therefore joint to identify novel chemical entities acting as DNPNB enzymes modulators.

### 5.2 IMPDH: drug development for human and bacteria, and inspection of all possible binding pockets

Among all DNPNB enzymes, IMPDH was the most scrupulously studied from a fundamental and therapeutic point of view in eukaryotes and prokaryotes.

**FIGURE 10:**
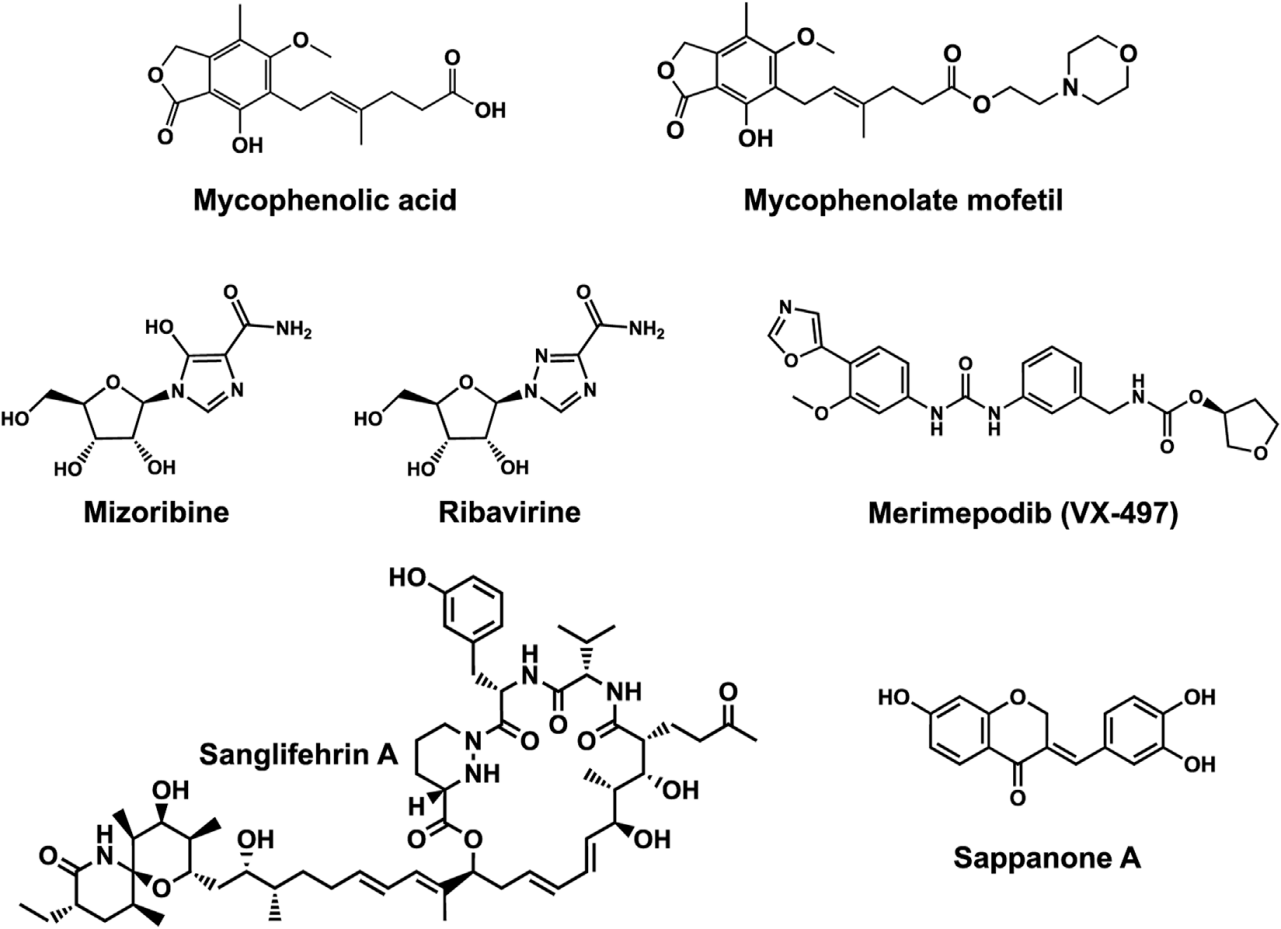
Some human IMPDH inhibitors.

Human IMPDHs have been of significant interest for the development of anticancer, immunosuppressive, and antiviral chemotherapies. Indeed, several molecules acting as competitive inhibitors for IMP or NAD^+^ were discovered and approved for use for the treatment of patients (Figure 10). Different reviews relate the development of IMPDH inhibitors (Pankiewicz and Goldstein, 2003; Hedstrom, 2009; Cuny et al., 2017), mainly for human counterparts. Historically, the first IMPDH inhibitor to be evaluated was mycophenolic acid [MPA; (Bentley, 2000)]. It was discovered in 1893 as a metabolite from penicillium fungi (Bentley, 2001), later characterized as an isobenzofuranone derivative and termed MPA (Alsberg and Black, 1913), and finally identified as an IMPDH inhibitor in 1969 (Franklin and Cook, 1969). MPA acts as an uncompetitive inhibitor by binding onto the nicotinamide moiety site in the active site of IMPDH and blocks the covalent [enzyme-XMP] complex (Hedstrom and Wang, 1990; Link and Straub, 1996). This molecule is a potent immunosuppressive that was approved by the FDA in 2004 (under the formulation mycophenolate sodium; Myfortic^®^, Novartis Inc.) and is administered in a tripartite regimen for patients as a prophylaxis for kidney transplants. Despite mixed reports detailing hematological and gastrointestinal toxicity or infectious susceptibility of MPA (Kiang and Ensom, 2019), pharmacological optimizations led to the synthesis of a more stable derivative prodrug, named mycophenolate mofetil (MPM) (CellCept^®^, Roche).

Nucleoside-based IMPDH inhibitors were also explored, as the example of mizoribine and ribavirin. Both mizoribine and ribavirin require metabolic activation to their corresponding 5′-monophosphates to compete for IMP. Mizoribine, also discovered as the case of MPA from a soil fungus, was approved in Japan (but not in the U.S.) as an immunosuppressant agent. Ribavirin is a synthetic molecule that was synthesized in 1972 (Witkowski et al., 1972) and revealed as a potent antiviral molecule against DNA and RNA viruses. However, this broad antiviral potency involves several mechanisms besides IMPDH inhibition and alterations of nucleotide pools. This molecule, first approved in 1998, is widely used in combinatory treatments of chronic hepatitis C infections.

Thanks to a structure-based drug design approach, Vertex Pharmaceuticals has developed merimepodib (VX-497) acting as an uncompetitive IMPDH inhibitor (Sintchak and Nimmesgern, 2000). It is an immunosuppressive agent (Jain et al., 2001). It also showed antiviral activity against several viruses, nonetheless, clinical development against hepatitis C (McHutchison et al., 2005) and SARS-CoV-2 (Arunachalam et al., 2020) were halted due to safety and efficiency concerns.

Other molecules acting as specific human IMPDH-inhibitors by binding to pockets distant from the active site have also been investigated. Two natural products (Figure 10), the polyketide/non-ribosomal peptide Sanglifehrin A, and the phenolic compound Sappanone A, were also revealed to be allosteric inhibitors of IMPDH. Sanglifehrin A exhibits immunosuppressive activity and binds to cyclophilin A and to the Bateman domain of hIMPDH2 (but not hIMPDH1) through the 22-membered macrocycle and the spirolactam cycle respectively. However, Sanglifehrin A does not alter hIMPDH2 enzymatic activity or *de novo* purine and pyrimidine biosynthesis in cells (Zenke et al., 2001; Pua et al., 2017), meaning that binding to IMPDH may possibly result in enzyme sequestration. Sappanone A acts as a hIMPDH2 selective inhibitor (Liao et al., 2017) by covalently binding to the thiol function of cysteine 140, a residue conserved in eukaryotic hIMPDH2 (but replaced by other residues in hIMPDH1 and bacterial orthologs). On a molecular level, Sappanone A inactivates hIMPDH2 catalytic activity, perturbs homooligomerization in cells, and exhibits potent anti-neuroinflammatory activity *in vivo*.

**FIGURE 11:**
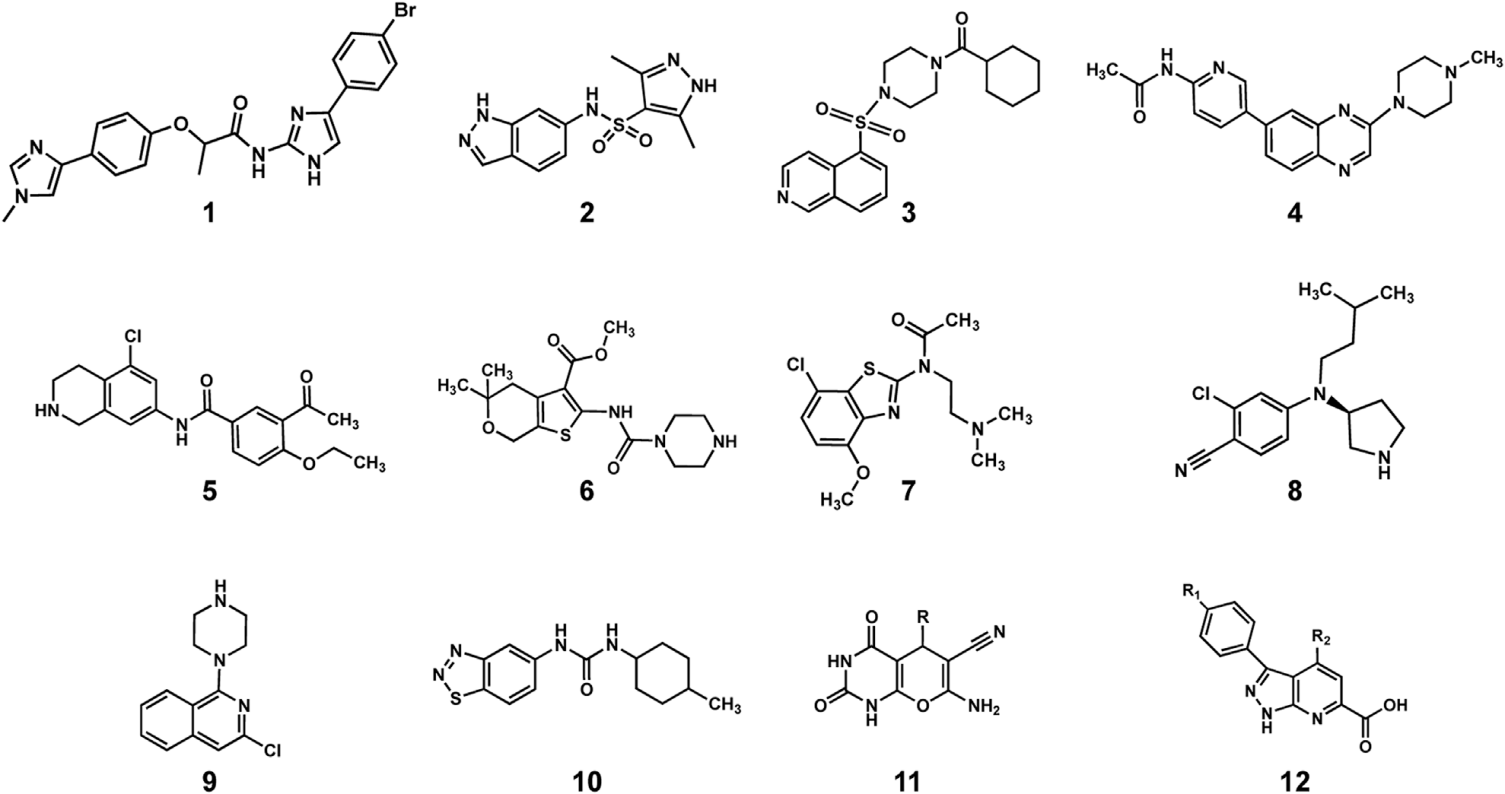
Novel chemical entities as potent bacterial IMPDH inhibitors. See text for references.

To date, no antibiotics targeting bacterial IMPDHs (or other DNPNB enzymes) have been advanced to clinical developments. As already mentioned in Section 5.1, IMPDH has been validated as a promising drug target for different bacterial pathogens such as *M. tuberculosis*, *P. aeruginosa* and *S. aureus*. On the other hand, the described human IMPDH inhibitors exhibit no or very low antibacterial potency (MPA being the best example of a eukaryotic specific one). However, this constitutes an argument in favor of the possibility of developing specific antibacterial compounds. IMPDH inhibitors specific for bacterial IMPDHs have been first described for a bacterial-like IMPDH [IMPDH from *Cryptosporidium parvum*; (Umejiego et al., 2004)]. Later, several research groups have investigated diverse scaffolds as specific IMP or NAD^+^-competitive inhibitors [see reviews: (Hedstrom et al., 2011; Shah and Kharkar, 2015; Cuny et al., 2017)]. More recently, considerable efforts have been concentrated on developing potent antituberculosis compounds (see Figure 11). Different novel chemical series have been explored after hit identification through either fragment-based [**1**; (Trapero et al., 2018)], phenotypic (**2**; (Park et al., 2017); and **3**; (Singh et al., 2017); or target-based whole cell [**4**–**10**; (Cox et al., 2016)] screenings. At the molecular level, all these compounds share a common feature as they are all bound within the catalytic domain (in the substrate binding pockets). In order to depart from the approaches listed above, Alexandre et al. (2019) have searched for allosteric inhibitors targeting the Bateman domain of IMPDH. Using a target-based approach on IMPDHpa, twelve hits have been identified as competitive inhibitors for ATP: nine of them were clustered into two chemical families (see Figure 11), namely, pyrano-[2,3]-pyrimidine (**11**) and pyrazolo-[3,4]-pyridine (**12**). Structural studies have revealed i) an original binding mode of these inhibitors (binding pocket composed of residues from two adjacent monomers, and only one binding site per monomer) and ii) that they trap the protein in a compact form of low affinity for IMP.

### 5.3 Targeting other DNPNB enzymes as novel therapeutic strategies

#### 5.3.1 Human enzymes

Other than IMPDH, TrifGART and ATIC have been targeted for the development of anticancerous agents. Both enzymes are linked to the one-carbon metabolism as they use N^10^-formyl FH_4_ as substrate. Different antifolates have been reported [see recent reviews (Dekhne et al., 2020; Cuthbertson et al., 2021)]. For instance, lometrexol has been identified as a specific TrifGART inhibitor (Taylor et al., 1985; Beardsley et al., 1989), but further developments were halted due to toxicity concerns. Structure-based drug design led to the identification of AG2034 that also showed unfortunate toxicity (Boritzki et al., 1996; Bissett et al., 2001) and AG 2037 (also called pelitrexol) which phase II clinical trials have been stopped because of a lack of sufficient efficacy when administered on its own. Another antifolate agent, named pemetrexed, was further developed, and approved by the FDA for the treatment of certain subtypes of lung cancer. This molecule, designated as « multi-targeted antifolate », acts as a broad inhibitor of not only TrifGART, but also ATIC and two other folate dependent enzymes, thymidylate synthase and dihydrofolate dehydrogenase (Shih et al., 1997). Recently, replacing the pyrrolo [2,3-d]pyrimidine core by a thieno [2,3-d]pyrimidine one led to the discovery of novel multitargeted inhibitors (Tong et al., 2023). The advantage of this series is their ability to be transported through folate receptors which confer a more selective delivery route to tumors compared to normal cells.

Apart these antifolates, cyclic peptides have been found to be potent ATIC inhibitors by impacting the dimerization of the enzyme (Tavassoli and Benkovic, 2005; Spurr et al., 2012). The most potent candidate (Cpd14; (Spurr et al., 2012); has been further used as a probe to get insight into the mode of activation of AMPK, since this peptide, by inhibiting ATIC dimerization, leads to increased intracellular ZMP level (Asby et al., 2015).

#### 5.3.2 Bacterial enzymes

In the case of bacterial enzymes, several compounds that display inhibition on specific bacterial DNPNB enzymes have been reported since the early 1990s. Chemical scaffolds are summarized in Table 3. Most of these molecules are substrate or cofactor analogues that act as competitive inhibitors for the active site.

**TABLE 3:** Summary of potential chemical scaffolds described as bacterial DNPNB enzymes inhibitors.

DNPNB target	Molecule	Reported activity	References
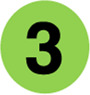 PurN	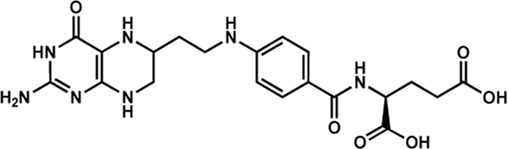 **13**	Folate analogue; competitive inhibitor for N^10^-formyl FH_4_ cofactor; IC_50_ = 0.1 µM on *Lactobacillus casei* PurN	Thorndike et al. (1990)
	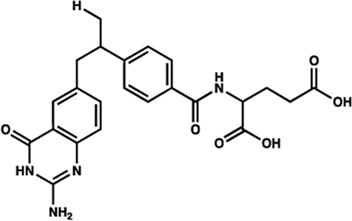 **14**	Folate analogue, competitive inhibitor for N^10^-formyl FH_4_ cofactor; K_i_ = 0.26 µM on *E. coli* PurN	Boger et al. (1997a), Boger et al. (1997b)
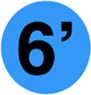 PurK	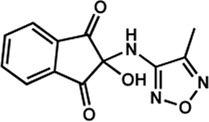 **15**	Class I inhibitor; IC_50_ = 2.3 µM on *E. coli* PurK; inhibition due to reaction with AIR and substrate depletion	Firestine et al. (2009)
	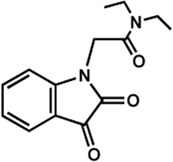 **16**	Class II inhibitor (isatin derivative); IC_50_ = 10.5 µM on *E. coli* PurK; inhibition due to AIR:isatin product formation and substrate depletion	Firestine et al. (2009), Streeter et al. (2019)
	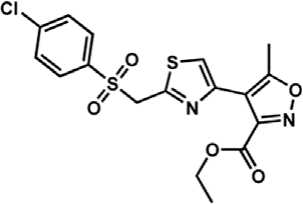 **17**	Class III inhibitor competitive for AIR, uncompetitive for ATP; IC_50_ = 10 µM on *E. coli* PurK	Firestine et al. (2009)
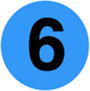 PurE	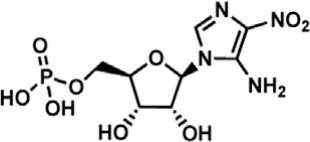 **18**	4-nitro-5-aminoimidazole ribonucleotide (NAIR), competitive inhibitor for CAIR; K_i_ = 0.5 µM on *E. coli* PurE	Firestine et al. (1994)
	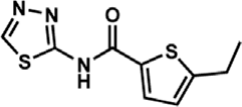 **19**	Fragment binder in the active site of *B. anthracis* PurE; K_D_ = 13.5 µM; 49% PurE inhibition at 25 µM	Lei et al. (2016)
	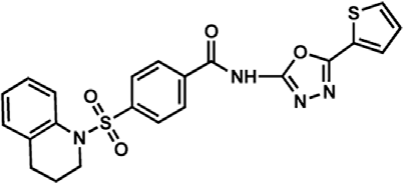 **20**	27% inhibition of *B. anthracis* PurE at 10 μM; MIC = 0.15, 0.3 and 0.4 μg/mL on *B. anthracis*, methicillin-susceptible and methicillin-resistant *S. aureus*, respectively	Kim et al. (2015a)
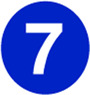 PurC	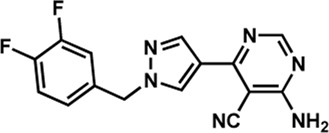 **21**	Binding site within the ATP-binding pocket; K_D_ = 0.15 µM on *M. abscessus* PurC; MIC = 50 µM on *M. tuberculosis*	Charoensutthivarakul et al. (2022)
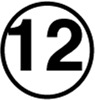 GuaA	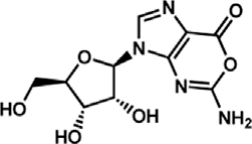 **22**	Oxanosine; K_i_ = 0.74 mM on *E. coli* GuaA; bacteriostatic on *E. coli*	Yagisawa et al. (1982)

For PurN, folate derivatives were explored and lead to several analogues (Thorndike et al., 1990; Boger et al., 1997a; Boger et al., 1997b), of which the two most potent are listed in Table 3 (**13** and **14**). However, a second target has been identified for both compounds although with a lower inhibitory potency (with 6 and 30 times increase in IC_50_ or K_i_ values for **13** and **14**, respectively): *L. casei* thymidylate synthase for **13** and another DNPNB enzyme for **14**, the avian bifunctional ATIC (impact on the AICART activity).

In the case of PurK, the studies of Firestine et al. (2009) revealed a series of inhibitors after a high-throughput screening (HTS) on the *E. coli* enzyme. The validated hits have been categorized into three classes: a representative of each class with the highest inhibitory potency is listed in Table 3 (**15**–**17**). The first class of compounds (indanedione core) has been shown to react with the substrate AIR. The second class (isatin derivatives) has been first described as being noncompetitive inhibitors for both substrates and considered as a promising chemical series. However, further characterization of their mode of action have shown that isatins react rapidly but reversibly with AIR, and therefore inhibit indirectly PurK by sequestering its substrate from the active site (Streeter et al., 2019). The third class is composed of inhibitors (such as **17**) selectively binding to the enzyme-ATP complex and thus inhibiting PurK activity.

For PurE, the first reported inhibitor was the CAIR analogue 4-nitro-5-aminoimidazole ribonucleotide (NAIR, **18**). This nucleotide was synthesized to get a better insight into the catalytic feature of PurE (Firestine and Davisson, 1993; Firestine et al., 1994). It is not a specific inhibitor of the *E. coli* enzyme, as it has also been shown to inhibit its avian counterpart. In 2006, Lei et al. (2016) have selected PurE from *B. anthracis* as a target, as it has been found essential for survival of this pathogen in serum (see above Section 5.1). Fragment library screenings have been performed by NMR and surface plasmon resonance (SPR). Different hits have been validated as binders in secondary assays, among them fragment **19**. This fragment showed the tightest binding affinities (K_D_ values determined by two different techniques in the 10 µM range) and the best inhibitory potency (around 50%) of the catalytic activity of *B. anthracis* PurE. On this same enzyme, a HTS of an antibacterial chemical library has been performed by thermal shift assay (Kim A. et al., 2015). Sixteen compounds have been further validated as inhibitors of *B. anthracis* PurE catalytic activity, although with moderate potencies (below 30% inhibition at 10 µM). However, some compounds (sharing the same core structure of 2-carboxamido-1,3,4-oxadiazole, such as **20**) were shown to be antimicrobials on different pathogens, including *B. anthracis*, *S. aureus* (methicillin-sensitive and resistant strains) and *F. tularensis*. The low MIC values (in the low µg/mL range and below) suggest that other protein(s) might be the target(s) of these compounds.

PurC, the next enzyme in the DNPNB (step 7), has also been considered as a promising antibacterial target. A fragment-based approach, guided by X-ray crystallography for the fragment growing step, has been applied on the *Mycobacterium abscessus* PurC (Charoensutthivarakul et al., 2022) leading to the identification of different 4-amino-6-(pyrazol-4-yl)pyrimidine derivatives with binding affinities in the low micromolar range (**21** exhibiting the lowest K_D_ value determined by isothermal titration calorimetry). Some of them also exhibit an antibacterial activity on *M. abscessus* and *M. tuberculosis* (MIC from 25 to 200 µM), but they need to be further developed to improve their potencies.

Finally, deciphering the mode of cation of oxanosine (Shimada et al., 1981), a nucleoside antibiotic (**22**), has led to the identification of its target in *E. coli*. It is a competitive inhibitor of GuaA for the substrate XMP, with a K_i_ value of 0.74 mM (Yagisawa et al., 1982). Besides its bacteriostatic activity, oxanosine has also been found to inhibit human cell growth but to be more cytotoxic to tumor than to normal cells (Uehara et al., 1985). Looking at the mammalian GMPS (the homologue of the bacterial GuaA), no impact of oxanosine, nor of its 5′-monophosphate derivative, has been observed on the catalytic activity. On the other hand, the monophosphate derivative has been found first to be a nearly competitive inhibitor with respect to IMP for rat IMPDH (Uehara et al., 1985). Later on, it was tested on different IMPDHs (including two bacterial ones, IMPDHba and IMPDHcj, and hIMPDH2) and demonstrated to be a competitive inhibitor for IMP (Yu et al., 2019). Investigating the inhibitory mechanism in more detail led to the conclusion by these same authors that a covalent adduct is formed with the catalytic cysteine of the enzyme.

### 5.4 Future perspectives

Antibiotic resistance remains a main concern of public health, and drugs acting with a novel mode of action are needed. Several possible strategies altering at least one of the regulatory modes of DNPNB listed in this review could be adopted to develop novel therapeutic agents. IMPDH is still to date the most attractive enzyme among all DNPNB enzymes in human and bacteria from a pharmacological point of view. Only few other bacterial DNPNB enzymes have been exploited as therapeutic targets, although most of them have been shown to play a crucial role in bacterial growth and virulence. Moreover, divergences exist between the bacterial and human DNPNB, therefore it would be possible to develop inhibitors acting only on pathogens, without any impact on humans. Some recent development for screening tools [such as the case of PurE, for example, as reported recently by Sharma et al. (2023)], has the potential to reignite interest in bacterial DNPNB enzymes and facilitate the discovery of novel chemical scaffolds. Nonetheless, despite their attractiveness, it is also worth noting that some possible limitations need to be kept in mind while developing DNPNB modulators. For example, perturbing the nucleotide pool equilibrium can possibly increase the rate of nonspecific incorporation of nucleotides during replication or translation, and therefore raise some serious pharmacodynamic issues (such as induction of mutations in housekeeping genes). Even though this concern is not completely applied to cancer cells, since targeting nucleotide metabolism has been shown to elicit significant therapeutic efficacy in cancer immunotherapies (Wu et al., 2022; Ali and Ben-Sahra, 2023; Mullen and Singh, 2023), this molecular phenomenon in bacteria can lead to the emergence and rise of multidrug-resistant strains.

Another approach could involve the development of modulators of protein-protein interactions (PPIs). In comparison with perturbators of enzymatic catalysis in which a molecule binds to the active site, allosteric or PPI modulators can be designed to be more specific to a certain bacterial or mammalian ortholog, since the allosteric and (homo/hetero)-oligomerization pockets are more evolutionarily divergent than the residues implicated in the binding of substrates and catalysis. Although PPIs have long been considered as challenging targets with small compounds, recent successful development of PPI inhibitors has been documented (Lu et al., 2020). This approach will also benefit from the availability of PPI-focused chemical libraries to increase the hit rate (Zhang et al., 2014; Bosc et al., 2020). With the discovery of the purinosome, and the recent updates supporting the existence of a DNPNB quinary structure in bacteria, several PPIs could be targeted by small molecules to alter protein colocalization and therefore DNPNB. This strategy was already described in the case of the bifunctional human ATIC, for example, with the report of a series of peptides that inhibit ATIC homodimerization (Tavassoli and Benkovic, 2005; Spurr et al., 2012). *In vitro* evaluation of one of the hits showed significant reduction of proliferation of breast cancer cells. In this perspective, it will be interesting to evaluate the effect of these molecules on purinosome formation in cells and DNPNB metabolic flux. Another enzyme of interest could be PurB, not only for its unique essentiality for bacterial growth, but also for its original three-dimensional structure. The resolution of *S. aureus* PurB structure has revealed that active sites are endowed between dimeric interfaces of the tetramer, and hence alteration of homooligomerization could potentially alter catalytic activity and protein-protein interactions (Fyfe et al., 2010).

Developing antibacterial compounds could be interesting not only for treatment of bacterial infections, but also to potentiate the effect of anticancer drugs. Attractively, it has been shown that the microbiome could be in some cases causative of chemoresistance in gastrointestinal and pulmonary cancers (Halley et al., 2020; Garajová et al., 2021). Developing dual inhibitors targeting human and/or bacterial DNPNB pathway and adding them to mixed therapies could be beneficial for some patients.

## Figures and Tables

**Table:** 

**ADP**	adenosine 5′-diphosphate
**AdS**	adenylosuccinate
**ADSL**	Ads lyase
**ADSS**	Ads synthase
**AICAR (or ZMP)**	5-aminoimidazole-4-carboxamide ribonucleotide
**AIR**	5-aminoimidazole ribonucleotide
**AMP**	adenosine 5′-monophosphate
**AMPK**	AMP-activated protein kinase
**ATIC**	bifunctional AICAR formyltransferase domain (**AICART**)/IMP cyclohydrolase domain (**IMPC**)
**ATP**	adenosine 5′-triphosphate
**CAIR**	5-phosphoribosyl-4-carboxy-5-aminoimidazole
**CBS**	cystathionine-β-synthase
**CK2**	casein kinase 2
**DNPNB**	*de novo* purine nucleotide biosynthesis
**FAICAR**	5-formamidoimidazole-4-carboxamide ribonucleotide
**FGAM**	5′-phosphoribosyl-N-formyl glycineamidine
**FGAMS**	FGAM synthase
**FGAR**	5′-phosphoribosyl-N-formylglycinamide
**FH**_**4**_	5,6,7,8-tetrahydrofolic acid
**GAR**	5′-phosphoribosylglycinamide
**GDP**	guanosine 5′-diphosphate
**GFP**	green fluorescent protein
**GMP**	guanosine 5′-monophosphate
**GMPR**	guanosine 5′-monophosphate reductase
**GMPS**	GMP synthase
**GPCRs**	G protein-coupled receptors
**GTP**	guanosine 5′-triphosphate
**hIMPDH1**	type 1 human IMPDH
**hIMPDH2**	type 2 human IMPDH
**Hsp**	heat-shock protein
**HTS**	high-throughput screening
**IMP**	inosine 5′-monophosphate
**IMPDH**	IMP dehydrogenase
**IMPDHag**	*Ashbya gossypii* IMPDH
**IMPDHbs**	*Bacillus subtilis* IMPDH
**IMPDHcj**	*Campylobacter jejuni* IMPDH
**IMPDHec**	*Escherichia coli IMPDH*
**IMPDHpa**	*Pseudomonas aeruginosa* IMPDH
**IMPDHsc**	*Streptomyces coelicolor* IMPDH
**IMPDHtb**	*Trypanosoma brucei* IMPDH
**MIC**	minimum inhibitory concentration
**MPA**	mycophenolic acid
**MPM**	mycophenolate mofetil
**MTHFD2**	methylenetetrahydrofolate dehydrogenase 2
**MTHFS**	methenyltetrahydrofolate synthetase
**mTORC1**	mammalian target of rapamycin complex 1
**NAD** ^**+**^ **/NADH**	nicotinamide adenine dinucleotide
**NAIR**	4-nitro-5-aminoimidazole ribonucleotide
**NCAIR**	N^5^-carboxyamino-imidazole ribonucleotide
**NMR**	nuclear magnetic resonance
**Nwd1**	NACHT and WD repeat domain containing protein 1
**OFP**	orange fluorescent protein
**PAICS**	bifunctional phosphoribosyl aminoimidazole carboxylase (**CAIRS**) and phosphoribosyl-aminoimidazole-succinocarboxamide synthase (**SAICARS**)
**PDK1**	3-phosphoinositide-dependent kinase-1
**PI-2P**	phosphatidylinositol (4,5)-bisphosphate
**PI-3P**	phosphatidylinositol (3,4,5)-trisphosphate
**PI3K**	phosphatidylinositol 3-kinase
**PKB (or Akt)**	protein kinase B
**PKC**	protein kinase C
**PPAT**	PRPP amidotransferase
**ppGp**	guanosine-3′-monophosphate-5′-diphosphate
**ppGpp**	guanosine-3′,5′-tetraphosphate
**pppGpp**	guanosine-3′-diphosphate-5′-triphosphate
**PPI**	protein-protein interaction
**PRA**	5-phosphoribosyl-1-amine
**PRPP**	5-phosphoribosyl-1-pyrophosphate
**PTMs**	post-translational modifications
**R**_**5**_ **P**	ribose 5-phosphate
**S6K**	p70 ribosomal S6 kinase
**SAICAR**	5-Amino-4-imidazole-N-succinocarboxamide ribonucleotide
**SAXS**	small-angle X-ray scattering
**TEADs**	TEA domain transcription factors
**TIM**	triose-phosphate isomerase
**TrifGART**	Trifunctional GAR synthetase (**GARS**), GAR formyltransferase (**GART**), AIR synthetase (**AIRS)** domains
**UTR**	untranslated region
**VGLL3**	vestigial-like family member 3
**XMP**	xanthosine 5′-monophosphate
